# Use of Tobacco Products and Suicide Attempts Among Elementary School–Aged Children

**DOI:** 10.1001/jamanetworkopen.2024.0376

**Published:** 2024-02-26

**Authors:** Phil H. Lee, Brenden Tervo-Clemmens, Richard T. Liu, Maia B. Gersten, Jae-Yoon Jung, Amy C. Janes, Jodi Gilman

**Affiliations:** 1 Department of Psychiatry, Massachusetts General Hospital and Harvard Medical School, Boston; 2 Center for Genomic Medicine, Massachusetts General Hospital, Boston; 3 Department of Psychiatry and Behavioral Sciences, University of Minnesota, Minneapolis; 4 Center for Addiction Medicine, Massachusetts General Hospital, Boston; 5 Depression Clinical and Research Program, Massachusetts General Hospital, Boston; 6 Division of Neuropsychiatry, Massachusetts General Hospital, Boston; 7 Department of Pediatrics, Stanford University, Stanford, California; 8 Cognitive and Pharmacological Neuroimaging Unit, National Institute on Drug Abuse, Biomedical Research Center, Baltimore, Maryland

## Abstract

**Importance:**

The use of tobacco products, including e-cigarettes and vaping, has rapidly increased among children. However, despite consistent associations found between smoking cigarettes and suicidal behaviors among adolescents and adults, there are limited data on associations between emerging tobacco products and suicidal behaviors, especially among preadolescent children.

**Objective:**

To examine whether the use of tobacco products is associated with nonsuicidal self-injury (NSSI), suicidal ideation (SI), and suicide attempts (SAs) among preadolescent children.

**Design, Setting, and Participants:**

This cohort study, conducted from September 1, 2022, to September 5, 2023, included participants in the Adolescent Brain Cognitive Development study, a population-based cohort of 11 868 US children enrolled at 9 and 10 years of age. The cross-sectional investigation focused on 3-year periods starting from the baseline to year 2 of follow-up. Statistical analysis was performed from October 1, 2022, to June 30, 2023.

**Main Outcomes and Measures:**

Children’s use of tobacco products was assessed based on youth reports, including lifetime experiences of various nicotine-related products, supplemented with hair toxicologic tests. Main outcomes were children’s lifetime experiences of NSSI, SI, and SAs, assessed using the K-SADS-5 (Kiddie Schedule for Affective Disorders and Schizophrenia for the *DSM-5*). Multivariate logistic regression was conducted to examine the associations of the use of tobacco products with NSSI, SI, and SAs among the study participants. Sociodemographic, familial, and children’s behavioral, temperamental, and clinical outcomes were adjusted in the analyses.

**Results:**

Of 8988 unrelated study participants (median age, 9.8 years [range, 8.9-11.0 years]; 4301 girls [47.9%]), 101 children (1.1%) and 151 children (1.7%) acknowledged lifetime use of tobacco products at baseline and at 18-month follow-up, respectively. After accounting for various suicide risk factors and potential confounders, children reporting use of tobacco products were at a 3 to 5 times increased risk of SAs (baseline: n = 153 [adjusted odds ratio (OR), 4.67; 95% CI, 2.35-9.28; false discovery rate (FDR)–corrected *P* < .001]; year 1: n = 227 [adjusted OR, 4.25; 95% CI, 2.33-7.74; FDR-corrected *P* < .001]; and year 2: n = 321 [adjusted OR, 2.85; 95% CI, 1.58-5.13; FDR-corrected *P* = .001]). Of all facets of impulsivity measures that were significant correlates of use of tobacco products, negative urgency was the only independent risk factor for SAs (adjusted OR, 1.52 [95% CI, 1.31-1.78]; FDR-corrected *P* < .001). In contrast, children’s alcohol, cannabis, and prescription drug use were not associated with SAs.

**Conclusions and Relevance:**

This study of US children suggests that the increased risk of SAs, consistently reported for adolescents and adults who smoke cigarettes, extends to a range of emerging tobacco products and manifests among elementary school–aged children. Further investigations are imperative to clarify the underlying mechanisms and to implement effective preventive policies for children.

## Key Points

**Question:**

Is the use of tobacco products associated with an increased risk of self-injurious thoughts and behaviors among children?

**Findings:**

This cohort study of 8988 preadolescent children enrolled in the Adolescent Brain Cognitive Development study found statistically significant associations between children’s use of tobacco products and suicide attempts. These associations were partially related to increased negative urgency and remained significant after accounting for various demographic, socioeconomic, familial, and clinical risk factors of suicide.

**Meaning:**

The findings suggest that smoking tobacco products may be a modifiable risk factor that can be addressed in suicide prevention efforts, especially among children.

## Teaser

This cohort study examines whether the use of tobacco products is associated with nonsuicidal self-injury, suicidal ideation, and suicide attempts among preadolescent children.

## Introduction

The increasing use of various tobacco products, including e-cigarettes, vapes, and hookahs, among children and adolescents is a critical public health problem worldwide.^1^ Smoking, especially when started at a young age, has been associated with lifelong negative mental health outcomes, ranging from diminished neurocognition^2,3,4^ to neurodevelopmental alterations^5,6,7,8,9,10^ to increased risk of delinquent behaviors and addiction^11^ in later life.^12,13,14^ Multiple population-based studies have reported that adolescent smokers exhibit a 2 to 5 times greater risk of self-injurious thoughts and behaviors (SITBs), even after adjusting for their mental health problems.^15,16,17^ Higher rates of suicide risk are consistently reported among current smokers compared with individuals who quit,^16,18,19,20,21^ underscoring that further investigations are necessary to clarify whether smoking could be a major preventable risk factor associated with suicidal behaviors.^22^

Compared with adolescents^15,16,17^ and adults,^21,23^ however, there is a dearth of information available on the association between smoking and suicidal behaviors among preadolescent children. Many observational studies focused on uncovering risk factors of childhood smoking initiation, including prenatal exposure,^24^ psychiatric disorders,^25^ family conflict,^24,26^ and peer influence.^5^ Several groups examined brain structures and functional networks for biomarkers of early-onset smoking.^5^ Others inspected genome-wide data to elucidate the genetic effects shared between smoking and childhood psychopathologic problems.^27^ Some of these same risk factors (eg, psychiatric disorders, family conflict or dysfunction, peer relationships) have similarly been associated with preadolescent suicidal thoughts and/or behavior.^28^ However, it remains an open question whether associations between smoking and suicidal behaviors, consistently observed among adolescents and adults who use combustible cigarettes, also manifest among preadolescent children,^29^ an increasing number of whom are exposed to emerging tobacco products.

The primary aim of this study was to fill in this major knowledge gap. We analyzed data from the Adolescent Brain Cognitive Neurodevelopment (ABCD) study, a US population-based cohort of 11 878 elementary school–aged children (enrolled at 9-10 years of age). The ABCD data consist of various social, familial, mental, and physical well-being and behavioral measures assessed from baseline and the first 2 years of follow-up (release version 4.0). Using these data, we examined the following 3 questions: (1) Is the use of tobacco products associated with SITBs among children, while accounting for demographic, familial, and socioeconomic confounders? (2) If so, are children’s cognition, temperament, and psychopathologic outcomes associated with the link between smoking and SITBs? (3) Are these findings unique to the use of tobacco products or shared with other substance use problems, such as alcohol, cannabis, and prescription drugs use, among children? This research should have an effect on the prioritization of smoking prevention and cessation efforts in health care policies, especially concerning suicide prevention among children and adolescents.

## Methods

### Study Participants

The ABCD study, an ongoing longitudinal study that began in 2017, enrolled a total of 11 868 participants aged 9 and 10 years in 22 US study sites (eTable 1 in Supplement 1). We downloaded ABCD study data, version 4.0, from September 1, 2022, to September 5, 2023, from the National Institute of Mental Health Data Archive. Comprehensive information regarding sample collection, survey measures, and study protocols is available elsewhere.^30,31,32^ The ABCD study was approved by a central institutional review board at the University of California, San Diego. All ABCD participants and their caregivers provided written informed assent and consent for human research. This study is the secondary analysis of deidentified ABCD participants and is exempt from informed consent by the Massachusetts General Hospital institutional review board. This report followed the Strengthening the Reporting of Observational Studies in Epidemiology (STROBE) reporting guideline.

### Measures of the Use of Tobacco Products

Children’s substance use data were assessed at baseline using the ABCD Youth Substance Use Interview.^29^ The ABCD Youth Mid-Year Phone Interview for Substance Use was conducted at the 6-month follow-up and then on a yearly basis, asking whether the child has smoked tobacco products at any time in the past 6 months until the interview date. We assembled hair toxicology screening test results from the ABCD Youth Hair Results. Using these data, we generated 3 binary variables for lifetime use of tobacco products corresponding to baseline, 6-month follow-up, and 18-month follow-up. Participants were classified as cases if they ever reported the use of tobacco products in the surveys or had positive test results for cotinine in the hair toxicology tests (eTable 2 in Supplement 1).

### Measures of Suicidal Behaviors

Data on SITBs of the participants were obtained from the youth and caregiver reports of the K-SADS-5 (Kiddie Schedule for Affective Disorders and Schizophrenia for the *DSM-5*). Lifetime nonsuicidal self-injuries (NSSIs), suicide attempts (SAs), and suicidal ideation (SI) were assessed based on the K-SADS-5 suicide survey module (eTable 3 in Supplement 1). Considering the young age of the ABCD study participants, SA cases included participants who reported attempted, interrupted, and/or aborted SA actions with intention to die, while SI cases were participants with passive or active thoughts of suicide but without any SA actions. Nonsuicidal self-injury cases were participants who reported NSSIs without reporting any SA or SI experience (eFigure 1 in Supplement 1). Response rates for the use of tobacco products and SITB outcomes are provided in eTable 4 in Supplement 1.

### Additional Measures on Family and Children

To identify and control for potential confounding factors, we examined demographic characteristics, socioeconomic characteristics, family history, and prenatal exposure of ABCD study participants stratified by their use of tobacco products status using the ABCD Parent Demographics Survey, Parent Family History Summary, and Developmental History Questionnaire (eTable 5 in Supplement 1). Parent-reported race and ethnicity information was obtained from the ABCD Parent Demographics Survey. For children’s cognition, temperament, and psychopathology outcomes, we analyzed data from the ABCD Child Behavior Checklist, National Institutes of Health Toolbox Summary Scores, Pearson Scores, Little Man Task Summary Scores, Mental Health Youth Sum Scores, and Early Adolescent Temperament Questionnaire Parent data (eTable 6 in Supplement 1). We generated ever-use experiences of each participant’s alcohol sipping or tasting, alcohol drinking (ie, at least a full cup of drinking), cannabis use, and prescription use at baseline, 6-month follow-up, and 18-month follow-up (eTable 7 in Supplement 1).

### Statistical Analysis

Statistical analysis was performed from October 1, 2022, to June 30, 2023. Bivariate analyses were conducted for summarizing the characteristics of the study participants stratified by their use of tobacco product status. We examined demographic characteristics (age, sex, parent-reported race and ethnicity [Asian, Black, Hispanic, White, and other (American Indian or Alaska Native, Native Hawaiian or Other Pacific Islander, multiple races, or other in the survey)]), socioeconomic factors (parental education, household income, parental marital status), parental history (depression, alcohol abuse, drug use, behavioral troubles, suicide), and prenatal exposure to smoking. Race and ethnicity information was included because smoking behaviors are influenced by cultural and social norms, which may vary among different racial and ethnic groups. Unpaired *t* tests were used for continuous variables, and χ^2^ tests were used for categorical variables. False discovery rates (FDRs) were calculated to ensure a proper type I error rate at 5% for all analyses involving multiple testing.

Multivariate logistic regression was conducted to examine the association of the use of tobacco products with NSSI, SI, and SAs. First, we examined a basic regression model, in which the use of tobacco products and each SITB outcome were tested as an independent and a dependent variable, respectively, while controlling for age, sex, and parent-reported race and ethnicity. In the following full covariate-adjusted model, we additionally included sociodemographic, family history, and prenatal substance use variables that were identified as significant correlates of the use of tobacco products in the preceding bivariate analyses. We also examined whether specific children’s cognition, temperaments, and psychopathology outcomes were significant correlates of the use of tobacco products using multivariate logistic regression. All child behavioral outcomes with significant correlation with the use of tobacco products were added in the full covariate-adjusted regression model to estimate the independent association of the use of tobacco products with SITBs above and beyond these potential confounders. Last, we evaluated the specificity of the association between the use of tobacco products and the risk of SITB outcomes by comparing the analysis results with other types of substance use. Statistical analysis was conducted in R, version 4.2.1 (R Project for Statistical Computing). All *P* values were from 2-sided tests, and results were deemed statistically significant at an FDR-corrected *P* < .05.

## Results

### Study Sample

Table 1 summarizes the major characteristics of ABCD participants stratified by their lifetime use of tobacco products at baseline. Of the 8988 unrelated youths (median age, 9.8 years [8.9-11.0]; 4301 girls [47.9%]) included in this study (eFigure 2 in Supplement 1), 101 (1.1%) reported ever using tobacco products at baseline. Of these participants, 42 girls (41.6%) reported the use of tobacco products (median age, 10.1 years [range, 8.2-11.0 years]). A total of 8887 youths (98.9%; 4259 girls [47.9%]; median age, 9.8 years [range, 8.9-11.0 years]) did not report use of tobacco products. Prevalence of smoking experience was not statistically different in either sex or across different races and ethnicities. However, children in the case group were exposed to more disadvantaged socioeconomic environments and smoking during pregnancy and were more likely to have parents with depression, alcohol problems, behavioral troubles, and suicide-related incidences compared with children in the control group. Although the prevalence of tobacco product use increased significantly from 1.1% (n = 101) at baseline to 1.7% (n = 151) at 18-month follow-up (χ^2^ = 9.66; *P* = .002), all major characteristics of the group that used tobacco products observed at baseline remained consistent in the follow-up years (eTables 8 and 9 in Supplement 1).

**Table 1.** Major Characteristics of Adolescent Brain Cognitive Development Study Participants Based on Youth-Reported Ever Use of Tobacco Products in the Baseline

Characteristic^b^	Ever use of tobacco products (baseline)^a^		
	Cases (n = 101 [1.1%])	Controls (n = 8887 [98.9%])	*P* value^c^
Age, median (range), y^d^	10.1 (8.9-11.0)	9.8 (8.9-11.0)	.09
Sex, No. (%)			
Male	59 (58.4)	4628 (52.1)	.24
Female	42 (41.6)	4259 (47.9)	
Race and ethnicity, No. (%)			
Asian	1 (1.0)	210 (2.4)	.009
Black	16 (15.8)	1221 (13.7)	
Hispanic	15 (14.9)	1752 (19.7)	
White	48 (47.5)	4785 (51.3)	
Other^e^	21 (20.8)	919 (10.3)	
Parental education, No. (%)			
<High school diploma or GED certification	6 (5.9)	345 (3.9)	<.001^f^
High school diploma or GED certification	16 (15.8)	759 (8.5)	
Some college	43 (42.6)	2269 (25.5)	
Bachelor’s degree	20 (19.8)	2301 (25.9)	
Postgraduate degree	16 (15.8)	3213 (36.3)	
Household income, No. (%)			
≤$50 000	45 (44.6)	2078 (23.4)	<.001^f^
>$50 000 and ≤$100 000	30 (29.7)	2328 (26.2)	
>$100 000	26 (25.7)	4481 (50.4)	
Marital status of parents, No. (%)			
Not married	55 (54.5)	2758 (31.0)	<.001^f^
Married	46 (45.5)	6129 (69.0)	
Parental history, No. (%)			
Alcohol problem	35 (34.7)	1282 (14.4)	<.001^f^
Drug problem	48 (47.5)	2685 (30.2)	<.001
Depression	33 (32.7)	945 (10.6)	<.001^f^
Behavioral troubles	33 (32.7)	1141 (12.8)	<.001^f^
Mental disorders	52 (51.5)	3519 (39.6)	.02
Suicide	17 (16.8)	477 (5.4)	<.001^f^
Prenatal smoking, No. (%)			
Before acknowledging pregnancy	45 (44.6)	1187 (13.4)	<.001^f^
After acknowledging pregnancy	25 (24.8)	459 (5.2)	<.001^f^

Abbreviation: GED, General Educational Development.

^a^ Cases represent children who acknowledged the use of tobacco products in the past or at present or had positive cotinine results from hair toxicology tests, while controls did not.

^b^ Information about the demographic, socioeconomic, and family history was obtained from the Adolescent Brain Cognitive Development Parent Demographic survey data.

^c^ *P* values were from χ^2^ tests for categorical variables (eg, sex) and unpaired *t* test for quantitative measures (eg, age).

^d^ Age in years represents the age in months of study participants at the time of the interview divided by 12.

^e^ Indicates study participants who selected either American Indian or Alaska Native, Native Hawaiian or Other Pacific, multiple races, or others in the Adolescent Brain Cognitive Development Parent Demographic survey.

^f^ Significant after multiple testing correction.

### Association of Smoking and SITBs

**Figure 1.**
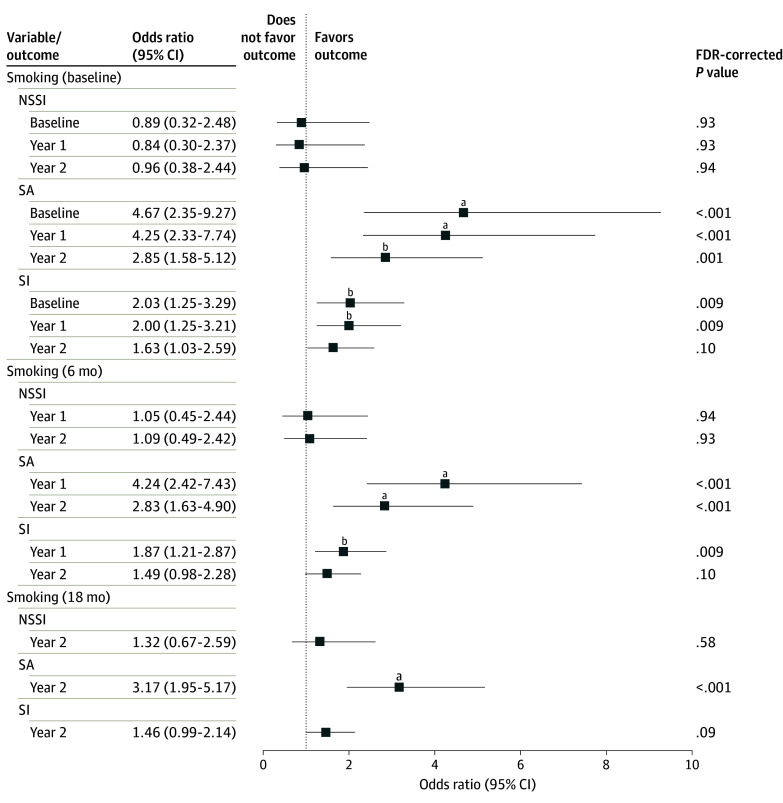
Associations of the Use of Tobacco Products With Suicide Risk Outcomes In multivariate logistic regression, lifetime use of tobacco products was used as an independent variable, along with other covariates, to measure associations with 3 suicide risk outcome measures: nonsuicidal self-injury (NSSI), suicide attempts (SAs), and suicidal ideation without SAs (SI). Fixed-effects covariates included age, sex, race and ethnicity, parents’ highest educational level, parents’ marital status, household income, prenatal exposure to smoking, parental history of depression, alcohol problems, behavioral troubles, and suicide. ^a^False discovery rate (FDR)–corrected *P* < .001. ^b^FDR-corrected *P* < .01.

First, we examined whether the use of tobacco products by children was associated with SITBs. Of 8988 youths, the lifetime prevalence of SITBs at baseline, year 1, and year 2, respectively, were as follows: NSSI, 4.8% (n = 428), 5.7% (n = 508), and 6.2% (n = 560); SI, 12.8% (n = 1151), 16.2% (n = 1454), and 20.6% (n = 1853); and SAs, 1.7% (n = 153), 2.5% (n = 227), and 3.6% (n = 321) (eFigure 1 in Supplement 1). In the basic model, we found statistically significant associations of the use of tobacco products with SAs and SI at all assessed time points (eFigure 3 and eTable 10 in Supplement 1). In the full covariate-adjusted model, the effect size of the use of tobacco products was attenuated, yet it remained independent and statistically significant for both SAs and SI (Figure 1; eTable 11 in Supplement 1). The use of tobacco products assessed at baseline was associated with SAs and SI at baseline, as well as with the outcomes reported in year 1 and year 2. Children reporting use of tobacco products were at a 3 to 5 times increased risk of SAs (baseline: n = 153 [adjusted odds ratio (OR), 4.67; 95% CI, 2.35-9.28; false discovery rate (FDR)–corrected *P* < .001]; year 1: n = 227 [adjusted OR, 4.25; 95% CI, 2.33-7.74; FDR-corrected *P* < .001]; and year 2: n = 321 [adjusted OR, 2.85; 95% CI, 1.58-5.13; FDR-corrected *P* = .001]). Similarly, lifetime use of tobacco products assessed at 6-month and 18-month follow-up was associated with the 2 SITB outcomes subsequently measured in year 1 and year 2. In all cases, the estimated effect sizes of the use of tobacco products for SAs were approximately 3 times larger compared with those for SI (Figure 1). Sensitivity analyses confirmed consistent findings across various measures of SITB outcomes (eTables 12-14 in Supplement 1). Despite limited power, we also found prospective associations of the use of tobacco products assessed at baseline and new SA cases reported in follow-up years 1 and 2 (ie, someone who did not report SAs at baseline but did in year 1 and year 2) (eTable 15 in Supplement 1). Conversely, we found no associations of the use of tobacco products with NSSI at all assessed times (Figure 1).

### Investigation of Children’s Cognition and Behavioral Measures

**Figure 2.**
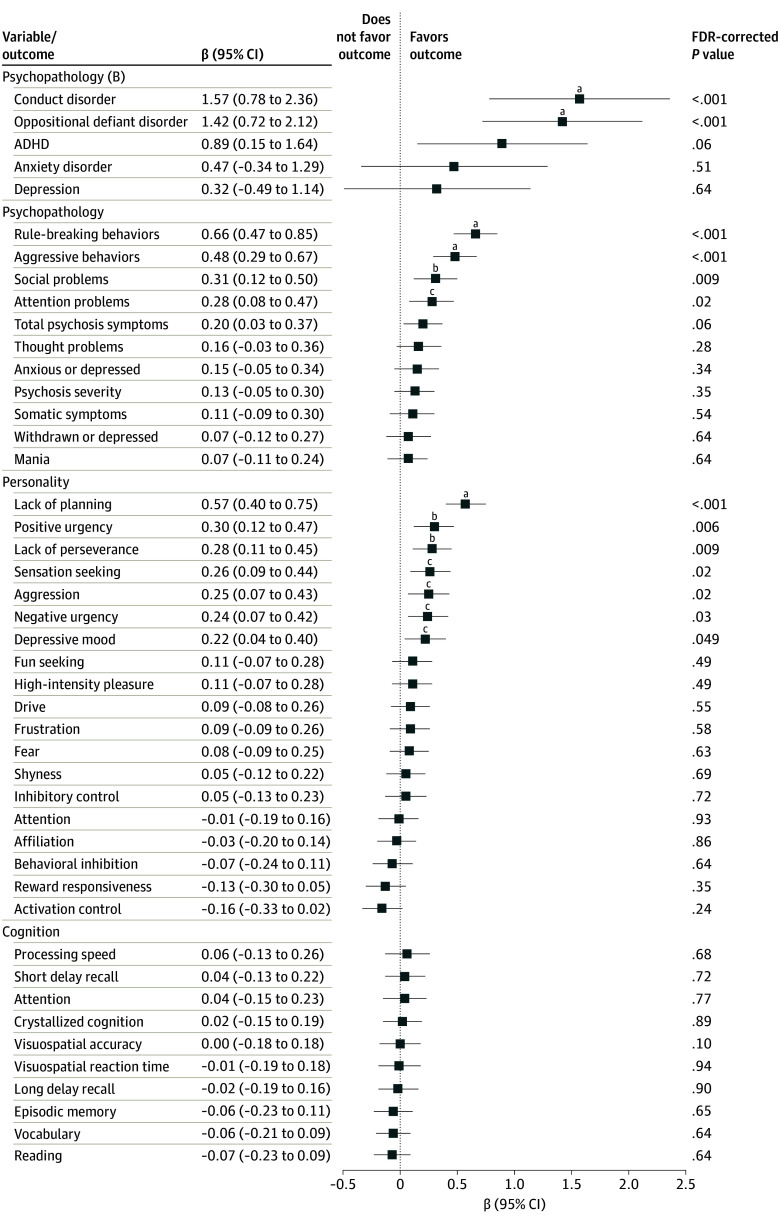
Associations of Children’s Personality, Cognition, and Psychology Outcomes With the Use of Tobacco Products The use of tobacco products was assessed in 18-month follow-up, while the assessed personality, cognition, and psychopathology outcomes were assessed in year 2. Effect size indicates the regression coefficient β of the exposure (ie, ever use of tobacco products) in multivariate regression, where each child outcome was used as a dependent variable. Additional covariates included age, sex, parent-reported race and ethnicity, parents’ highest educational level, household income, parents’ marital status, prenatal exposure to tobacco, number of substances used during pregnancy by mother, parental history of depression, alcohol problem, and behavioral troubles. The category Psychopathology (B) includes binary variables for *DSM-5*-–oriented Adolescent Brain Cognitive Development study Child Behavior Checklist measures. ADHD indicates attention-deficit/hyperactivity disorder. ^a^False discovery rate (FDR)–corrected *P* < .001. ^b^FDR-corrected *P* < .01. ^c^FDR-corrected *P* < .05.

Next, we examined whether the associations between the use of tobacco products and increased risk of SAs and SI are due to children’s cognition, temperament, and psychopathology outcomes. Of the 48 measures that we examined in year 2 (eTable 6 in Supplement 1), 13 were significant correlates of the use of tobacco products after multiple testing correction (Figure 2). Children who had reported the use of tobacco products at 18 months were more likely to have conduct disorder and oppositional defiant disorder compared with controls in year 2. These children also exhibited increased levels of rule-breaking behaviors, aggressive behaviors, and social problems relative to controls. In addition, there were notable differences in several impulsivity traits, including a lack of planning, positive urgency, and negative urgency.

However, both bivariate and multivariate regression analyses, which examined the 13 correlates of the use of tobacco products in the same model, found that the association between the use of tobacco products and SAs was independent of all childhood behavioral or psychopathology outcomes (OR, 3.09 [95% CI, 1.70-5.65]; *P* < .001; likelihood ratio test FDR-corrected *P* < .001) (Table 2). Negative urgency was the only correlate of the use of tobacco products that retained a significant association with SAs independent from other variables (OR, 1.52 [95% CI, 1.31-1.78]; *P* < .001; FDR-corrected *P* < .001). Smoking data assessed at baseline and 6-month follow-up presented similar findings (eTables 16 and 17 in Supplement 1). In contrast, the association between the use of tobacco products and SI dissipated once childhood behavioral measures were accounted for (eTables 18-20 in Supplement 1). Interaction analyses of smoking with childhood outcome measures did not reveal any significant associations with suicide risk outcomes (eTable 21 in Supplement 1).

**Table 2.** Multivariate Logistic Regression Results in Year 2

Independent variable	Multivariate logistic regression		Likelihood ratio test^a^		
	OR (95% CI)	*P* value	*R*^2^, %	*P* value	FDR-corrected *P* value
Demographic characteristics					
Age	1.13 (0.98-1.32)	.10	0.20	.10	.41
Female	0.91 (0.67-1.22)	.05	0.03	.52	.88
Race and ethnicity					
Asian	1.50 (0.52-4.34)	.04	0.00	>.99	>.99
Black	1.47 (0.91-2.36)	.12	0.00	>.99	>.99
Hispanic	1.40 (0.94-2.09)	.10	0.00	>.99	>.99
Other^b^	1.32 (0.84-2.07)	.24	0.00	>.99	>.99
Socioeconomic factors					
Income	1.06 (0.87-1.29)	.58	0.02	.06	.90
Married	0.59 (0.42-0.83)	.003	0.65	.003^c^	.03
Parental education					
High school diploma or GED certification	1.57 (0.67-3.67)	.30	0.00	>.99	>.99
Some college	1.76 (0.81-3.83)	.15	0.00	>.99	>.99
Bachelor’s degree	1.45 (0.63-3.36)	.38	0.00	>.99	>.99
Postgraduate	1.27 (0.53-3.03)	.59	0.00	>.99	>.99
Prenatal substance exposure					
Early tobacco exposure	0.89 (0.48-1.63)	.70	0.01	.70	.97
No early tobacco exposure	1.08 (0.88-1.32)	.44	0.04	.44	.85
Late tobacco exposure	1.37 (0.54-3.45)	.51	0.03	.51	.88
No late tobacco exposure	0.96 (0.78-1.19)	.72	0.01	.72	.97
Parental history					
Alcohol problems	0.86 (0.57-1.28)	.45	0.04	.45	.85
Depression	1.05 (0.67-1.67)	.82	0.00	.82	>.99
Behavioral troubles	1.14 (0.74-1.76)	.54	0.03	.55	.90
Suicide	1.72 (1.07-2.76)	.02	0.34	.03^c^	.27
Child psychopathology					
Rule-breaking behaviors	1.17 (1.00-1.37)	.05	0.27	.05	.34
Social problems	1.13 (0.98-1.32)	.10	0.19	.10	.41
Aggressive behaviors	1.10 (0.92-1.33)	.30	0.08	.30	.74
Attention problems	1.03 (0.89-1.19)	.73	0.01	.73	.97
Anxious or depressed	1.11 (0.94-1.30)	.21	0.11	.21	.59
Withdrawn or depressed	1.08 (0.93-1.25)	.30	0.07	.31	.74
*DSM-5* disorders					
Conduct disorder	0.72 (0.31-1.65)	.44	0.04	.44	.85
Oppositional defiant disorder	0.55 (0.26-1.18)	.12	0.18	.12	.42
Temperament					
Negative urgency	1.52 (1.31-1.78)	<.001	1.99	<.001^c^	<.001
Lack of planning	1.17 (1.00-1.37)	.05	0.26	.06	.34
Positive urgency	1.13 (0.98-1.32)	.10	0.19	.10	.41
Lack of perseverance	1.07 (0.92-1.25)	.37	0.06	.38	.84
Sensation seeking	0.90 (0.77-1.06)	.20	0.12	.20	.59
Aggression	0.97 (0.84-1.12)	.69	0.01	.69	.97
Depressive mood	0.90 (0.78-1.05)	.19	0.12	.19	.59
Substance use					
Use of tobacco products	3.09 (1.70-5.65)	<.001	0.83	<.001^c^	.011

Abbreviations: *DSM-5*, *Diagnostic and Statistical Manual of Mental Disorders* (Fifth Edition); FDR, false discovery rate; GED, General Educational Development; OR, odds ratio.

^a^ All listed independent variables were examined simultaneously in the regression model using suicide attempts as a dependent variable. Likelihood ratio tests were conducted by comparing the full regression model with the same model without the corresponding independent variable.

^b^ Indicates study participants who selected either American Indian or Alaska Native, Native Hawaiian or Other Pacific Islander, multiple races, or other in the Adolescent Brain Cognitive Development Parent Demographics survey.

^c^ Likelihood ratio test FDR less than 5%.

### Comparison With Other Substance Use Data

To evaluate the specificity of the association between the use of tobacco products and increased risk of SAs, we examined the association of SAs and smoking with other types of substance use data. Along with alcohol, cannabis, and prescription drug use, significant associations between SAs and use of tobacco products remained robust (use of tobacco products and alcohol sipping: OR, 2.70 [95% CI, 1.46-4.99]; FDR-corrected *P* = .003; use of tobacco products and alcohol drinking: OR, 3.26 [95% CI, 1.77-5.98]; FDR-corrected *P* = .008; use of tobacco products and cannabis use: OR, 2.78 [95% CI, 1.50-5.16]; FDR-corrected *P* = .04; and use of tobacco products and prescription drug use: OR, 2.89 [95% CI, 1.56-5.35]; FDR-corrected *P* = .03) (eTable 22 in Supplement 1). Of other substance use data, only experiences of alcohol sipping were associated with SAs independent of other variables (OR, 1.55 [95% CI, 1.14-2.13]; FDR-corrected *P* = .007).

## Discussion

Despite a rapid increase in the use of various tobacco products among youths, there have been limited data on its associations with mental health and suicidal behaviors among younger children. Using 3-year assessment data from a population-based sample of 8988 US preadolescent children, our study provides robust evidence that the use of tobacco products is associated with a 3 to 5 times increased risk of SAs during preadolescence. The heightened risk was detected among children as young as 9 and 10 years and manifested consistently in 2 subsequent years (aged <13 years).This association remained independent of well-established suicide risk factors, including sociodemographic, familial, and children’s psychopathology outcomes, as well as other types of substance use (including alcohol, cannabis, and prescription drugs).

Our study advances the field of pediatric suicide research in several important ways. First, to our knowledge, our findings provide the first empirical evidence that an increased risk of SAs, consistently reported for cigarette smokers,^15,16,17^ extends to a range of emerging tobacco products and was observable among preadolescent children. In recent years, e-cigarettes, vapes, and flavored nicotine products have gained wide popularity, in part because they are often marketed as safe alternatives to conventional cigarettes.^18,33,34^ However, several nationwide surveys have reported an increased risk of SAs among middle school and high school students with a lifetime use of e-cigarettes^16,35,36^ and vaping devices.^37,38,39,40^ Additional work suggests that the association between smoking and SAs may be largest for smoking initiation,^41^ which is consistent with the relatively early developmental patterns observed in the present work.

Second, our data extend prior evidence that use of tobacco products by children is a risk factor specifically associated with SAs but not with NSSI or SI. Using data from a community sample of 1458 youths aged 9 to 17 years, Wu et al^17^ reported significant associations between cigarette smoking and SAs after controlling for depression, while the previously significant association with SI disappeared. A study by Orri et al^42^ reported an association of prolonged exposure to maternal smoking during pregnancy and late childhood with increased risk of children’s SAs after accounting for various socioeconomic factors and parents’ mental health problems. Likewise, Orri et al^42^ did not find associations of maternal smoking with SI. The present study provides additional evidence for this distinction because the association between the use of tobacco products and children’s SAs remained robust after accounting for various confounders (including children’s own externalizing and internalizing problems), while the association with SI vanished. Our findings thus suggest that information on the use of tobacco products may help distinguish individuals who have SI from those who may act on their thoughts.^20,43,44^

Third, our data suggest that use of tobacco products was correlated specifically with externalizing problems and temperament issues rather than internalizing problems among this age group. For adolescents and adults, there have been mixed findings about the associations between suicidal behaviors, substance abuse, and psychiatric disorders. Several previous studies noted that smoking among youths with suicidal behaviors is likely to be secondary to affective disorders.^45,46^ Other groups reported that the associations between smoking and SAs were independent of depression and excessive alcohol use among adolescents and adults.^21,47,48^ Additional work has suggested a particular association with impulsive SAs that is more specific to externalizing symptoms.^49,50^

Our findings provide evidence that negative urgency is associated with both smoking exposure and increased risk of SAs among preadolescent children. We found several childhood behavioral outcomes that were significant correlates of the use of tobacco products, many of which, if not all, are established risk factors for substance use problems during adolescence.^51^ However, negative urgency was the only correlate of the use of tobacco products that remained an independent correlate of SAs after controlling for various suicide risk factors. The robust association between negative urgency and SAs is notable for several reasons. First, this association may be particularly relevant to this age group. This hypothesis is supported by age-related decreases in impulsivity from youth to adulthood^52,53^ and evidence that the association between impulsivity and suicide is stronger among youths than adults.^54,55^ Second, the finding that negative urgency specifically, rather than trait impulsivity more broadly, was a correlate of SAs is consistent with current conceptualizations of suicide risk. Several models of suicide^56,57^ posit that proximal risk for this outcome often occurs within circumscribed periods of high arousal. Further work is required to understand the neural mechanisms contributing to the association between smoking, negative urgency, and SAs.

### Limitations

Our study should be interpreted with several limitations in mind. First, although the ABCD study enrolled participants from various sites across the US, representing diverse geographical regions and demographic groups, the sample may not fully represent the entire US population.^30,31,32^ Findings from our study thus may not be generalizable to the entire US population of preadolescent children. Second, while we have identified significant associations between the use of tobacco products and SAs both cross-sectionally (at baseline) and prospectively (between different assessment events), our observational study design precludes the determination of causal relationships. For our additional thoughts on potential underlying mechanisms between the use of tobacco products and SAs, please refer to the eAppendix in Supplement 1). Third, we used hair toxicology tests to assess tobacco product use, which, while valuable, has limitations in detecting certain nicotine substances and may not capture the full range of tobacco exposure. Environmental factors, particularly exposure to secondhand smoke, might also be associated with the concentrations of substances detected in hair samples, therefore falsely increasing the prevalence of smoking among the study participants. Fourth, given the young age of the study participants, the prevalence of the use of tobacco products was less than 2%, limiting the statistical power to perform fine-grained secondary analyses, such as investigating the effect of individual types of tobacco products, the role of substance use patterns (eg, quantity, frequency, concentrations, administration routes), and proximal associations between newly developed use of tobacco products and SITBs between various assessment periods. As the ABCD study continues to collect data from adolescents over time, we anticipate that future datasets may offer improved statistical power to enhance our understanding of these critical aspects. Fifth, despite our efforts to control for various confounding factors, including sociodemographic variables, familial influences, and children’s psychopathology, the possibility of unmeasured confounders remains, which could influence both tobacco use and suicidal behaviors.

## Conclusions

In this study, children reporting tobacco use had an increased risk of SAs but not NSSI or SI. The findings suggest that smoking tobacco products may be a modifiable risk factor for suicide. Taken together, our findings suggest that the presumption that noncombustible tobacco products, including e-cigarettes and vapes, are safer alternatives to conventional cigarettes needs to be more thoroughly investigated,^58^ especially in the context of their potential effect on the mental health and suicidal behaviors of children and adolescents. Further research using neurobiology, genomics, and neuroimaging data is warranted to clarify the causal mechanisms underlying the association between the use of tobacco products and the increased risk of SAs through a developmental perspective.^59^ Until we have a clear understanding of the role of smoking and associated neural mechanisms, the use of tobacco products, particularly among children and adolescents, should not be overlooked. In conjunction with more active smoking prevention and intervention effort, we call for routine screening of the use of tobacco products among children and adolescents, especially when assessing suicide risk.

## Notes

Supplement 1. **eAppendix.** Discussion of Hypothesized Neurobiological Pathways and Shared Risk Factors Linking Smoking and Suicidal Behaviors **eFigure 1.** Number of Study Participants who Endorsed Suicide Attempts (SAs), Ideation (SI), and Non-Suicidal Self-Injuries (NSSI) from the KSADS-5, and Case Group Classifications Used in the Present Study **eFigure 2.** Flowchart for Study Participant Selection **eFigure 3.** Association Between the Use of Tobacco Products (UTPs) and Suicide Risk Outcomes Based on the Basic Model **eTable 1.** List of 22 ABCD Study Enrollment Sites **eTable 2.** Details of Children’s Tobacco Use Outcome Measures **eTable 3.** Details of KSADS-5 Variables Used for Constructing Lifetime Suicide Risk and Self-Injury Outcome Measures **eTable 4.** Response Rates for UTP and SITB Measures **eTable 5.** Data on Sociodemographics, Family History, and Prenatal Exposure to Substance Use **eTable 6.** Child Behavioral Outcome Measures **eTable 7.** Details of Children’s Substance Use Measures **eTable 8.** Major Characteristics of ABCD Study Participants Based on Youth-Reported Ever Use of Tobacco Products in the 6-Month Follow-Up **eTable 9.** Major Characteristics of ABCD Study Participants Based on Youth-Reported Ever Use of Tobacco Products in the 18-Month Follow-Up **eTable 10.** Association Between UTPs and Suicide Risk Outcomes Based on the Basic Model **eTable 11.** Association Between UTPs and Suicide Risk Outcomes Based on the All Covariate-Adjusted Model **eTable 12.** Association Between UTPs and KSADS-5 Youth-Report-Based Suicide Risk Outcomes Based on the All Covariate-Adjusted Model **eTable 13.** Association Between UTPs and KSADS-5 Parent-Report-Based Suicide Risk Outcomes Based on the All Covariate-Adjusted Model **eTable 14.** Association Between UTPs and Suicide Risk Outcome Measures Concordant Between KSAS-5 Youth- and Parent-Reports Assessed in the All Covariate-Adjusted Model **eTable 15.** Associations of the Use of Tobacco Products (UTPs) and New SA Cases, While Adjusting for Various Confounding Factors **eTable 16.** Multivariate Logistic Regression Results for Predicting Suicide Attempts Using UTPs (Both Lifetime Measures Assessed in the Baseline) **eTable 17.** Multivariate Logistic Regression Results for Predicting Suicide Attempts Using UTPs (UTPs Assessed in 6-Month Follow-Up, SA Assessed in Year 1 Follow-Up) **eTable 18.** Multivariate Logistic Regression Results for Predicting Suicidal Ideation Using UTPs (UTPs and SI Both Assessed in Baseline) **eTable 19.** Multivariate Logistic Regression Results for Predicting Suicidal Ideation Using UTPs (UTPs Assessed in 6-Month Follow-Up, SI Assessed in Year 1 Follow-Up) **eTable 20.** Multivariate Logistic Regression Results for Predicting Suicidal Ideation using UTPs (UTPs Assessed in 18-Month Follow-Up, SI Assessed in Year 2 Follow-Up) **eTable 21.** Interaction Analysis Results of UTP at Baseline with Children’s Cognition, Temperament/Personality, and Psychopathology Measures on SA. **eTable 22.** Multivariate Logistic Regression Results for Predicting Suicide Attempts (SAs) While Integrating Multiple Types of Substance Use Data in Addition to Smoking Tobacco Products

Supplement 2.
Data Sharing Statement
