# Supplementary material for: Use of Tobacco Products and Suicide Attempts Among Elementary School–Aged Children
Source: JAMA Netw Open. 2024 Feb 26;7(2):e240376. doi: 10.1001/jamanetworkopen.2024.0376 (PMC10897745; doi:10.1001/jamanetworkopen.2024.0376)
Supplement: Supplement 1. — eAppendix. Discussion of Hypothesized Neurobiological Pathways and Shared Risk Factors Linking Smoking and Suicidal Behaviors eFigure 1. Number of Study Participants who Endorsed Suicide Attempts (SAs), Ideation (SI), and Non-Suicidal Self-Injuries (NSSI) from the KSADS-5, and Case Group Classifications Used in the Present Study eFigure 2. Flowchart for Study Participant Selection eFigure 3. Association Between the Use of Tobacco Products (UTPs) and Suicide Risk Outcomes Based on the Basic Model eTable 1. List of 22 ABCD Study Enrollment Sites eTable 2. Details of Children’s Tobacco Use Outcome Measures eTable 3. Details of KSADS-5 Variables Used for Constructing Lifetime Suicide Risk and Self-Injury Outcome Measures eTable 4. Response Rates for UTP and SITB Measures eTable 5. Data on Sociodemographics, Family History, and Prenatal Exposure to Substance Use eTable 6. Child Behavioral Outcome Measures eTable 7. Details of Children’s Substance Use Measures eTable 8. Major Characteristics of ABCD Study Participants Based on Youth-Reported Ever Use of Tobacco Products in the 6-Month Follow-Up eTable 9. Major Characteristics of ABCD Study Participants Based on Youth-Reported Ever Use of Tobacco Products in the 18-Month Follow-Up eTable 10. Association Between UTPs and Suicide Risk Outcomes Based on the Basic Model eTable 11. Association Between UTPs and Suicide Risk Outcomes Based on the All Covariate-Adjusted Model eTable 12. Association Between UTPs and KSADS-5 Youth-Report-Based Suicide Risk Outcomes Based on the All Covariate-Adjusted Model eTable 13. Association Between UTPs and KSADS-5 Parent-Report-Based Suicide Risk Outcomes Based on the All Covariate-Adjusted Model eTable 14. Association Between UTPs and Suicide Risk Outcome Measures Concordant Between KSAS-5 Youth- and Parent-Reports Assessed in the All Covariate-Adjusted Model eTable 15. Associations of the Use of Tobacco Products (UTPs) and New SA Cases, While Adjusting for Various Confounding Factors eTable [file jamanetwopen-e240376-s001.pdf]

Supplementary Online Content

Lee PH, Tervo-Clemmens B, Liu RT, et al. Use of tobacco products and suicide attempts among elementary school–aged children. *JAMA Netw Open.* 2024;7(2):e240376. doi:10.1001/jamanetworkopen.2024.0376

eAppendix. Discussion of Hypothesized Neurobiological Pathways and Shared Risk Factors Linking Smoking and Suicidal Behaviors
eFigure 1. Number of Study Participants who Endorsed Suicide Attempts (SAs), Ideation (SI), and Non-Suicidal Self-Injuries (NSSI) from the KSADS-5, and Case Group Classifications Used in the Present Study
eFigure 2. Flowchart for Study Participant Selection
eFigure 3. Association Between the Use of Tobacco Products (UTPs) and Suicide Risk Outcomes Based on the Basic Model
eTable 1. List of 22 ABCD Study Enrollment Sites
eTable 2. Details of Children's Tobacco Use Outcome Measures
eTable 3. Details of KSADS-5 Variables Used for Constructing Lifetime Suicide Risk and Self-Injury Outcome Measures
eTable 4. Response Rates for UTP and SITB Measures
eTable 5. Data on Sociodemographics, Family History, and Prenatal Exposure to Substance Use
eTable 6. Child Behavioral Outcome Measures
eTable 7. Details of Children's Substance Use Measures
eTable 8. Major Characteristics of ABCD Study Participants Based on Youth-Reported Ever Use of Tobacco Products in the 6-Month Follow-Up
eTable 9. Major Characteristics of ABCD Study Participants Based on Youth-Reported Ever Use of Tobacco Products in the 18-Month Follow-Up
eTable 10. Association Between UTPs and Suicide Risk Outcomes Based on the Basic Model
eTable 11. Association Between UTPs and Suicide Risk Outcomes Based on the All Covariate-Adjusted Model
eTable 12. Association Between UTPs and KSADS-5 Youth-Report-Based Suicide Risk Outcomes Based on the All Covariate-Adjusted Model
eTable 13. Association Between UTPs and KSADS-5 Parent-Report-Based Suicide Risk Outcomes Based on the All Covariate-Adjusted Model
eTable 14. Association Between UTPs and Suicide Risk Outcome Measures Concordant Between KSAS-5 Youth- and Parent-Reports Assessed in the All Covariate-Adjusted Model
eTable 15. Associations of the Use of Tobacco Products (UTPs) and New SA Cases, While Adjusting for Various Confounding Factors
eTable 16. Multivariate Logistic Regression Results for Predicting Suicide Attempts Using UTPs (Both Lifetime Measures Assessed in the Baseline)
eTable 17. Multivariate Logistic Regression Results for Predicting Suicide Attempts Using UTPs (UTPs Assessed in 6-Month Follow-Up, SA Assessed in Year 1 Follow-Up)
eTable 18. Multivariate Logistic Regression Results for Predicting Suicidal Ideation Using UTPs (UTPs and SI Both Assessed in Baseline)
eTable 19. Multivariate Logistic Regression Results for Predicting Suicidal Ideation Using UTPs (UTPs Assessed in 6-Month Follow-Up, SI Assessed in Year 1 Follow-Up)
eTable 20. Multivariate Logistic Regression Results for Predicting Suicidal Ideation using UTPs (UTPs Assessed in 18-Month Follow-Up, SI Assessed in Year 2 Follow-Up)
eTable 21. Interaction Analysis Results of UTP at Baseline with Children's Cognition, Temperament/Personality, and Psychopathology Measures on SA.
eTable 22. Multivariate Logistic Regression Results for Predicting Suicide Attempts (SAs) While Integrating Multiple Types of Substance Use Data in Addition to Smoking Tobacco Products

This supplementary material has been provided by the authors to give readers additional information about their work.

© 2024 Lee PH et al. *JAMA Network Open.*

eAppendix. Discussion of Hypothesized Neurobiological Pathways and Shared Risk Factors Linking Smoking and Suicidal Behaviors

There are several plausible neurobiological pathways which may explain the relationship between UTPs and suicidal behaviors. Nicotine exposure affects brain plasticity, particularly in regions associated with emotion and memory (Levine et al. 2011; Huang et al. 2013; Kandel & Kandel, 2014), and can disrupt multiple biological systems, including those related to mood regulation and inflammation (Pandey, 2013, Rom et al. 2013). Notably, experssion of the brain-derived neurotrophic factor (BDNF), which is implicated in smoking initiation, is regulated by nicotine use and is also related to impulsivity and decision-making (Bhang et al. 2010; Jamal et al. 2015; Zhang et al. 2016)—traits linked to suicide (Kirch et al. 1987; Oquendo et al. 2014). Nicotine exerts direct effects on dopaminergic and glutamatergic neurotransmitter systems, which play critical roles in impulsivity, which is observed to increase with nicotine exposure, normalize upon cessation, and recur upon re-exposure (Kayir et al., 2014; Kolokotroni et al., 2014). Finally, alterations in synaptic plasticity in the brain's reward networks (Durazzo et al. 2010; van Heeringen & Mann, 2014), as well as serotonergic and receptor-linked signaling (Malone et al. 2003), may explain the relationship between UTPs and increased risk for suicide. Last but not the least, nicotine and suicide may share common risk factors encompassing genes and environmental stressors.

eReferences.

Levine A, Huang Y, Drisaldi B, Griffin Jr. EA, Pollak DD, Xu S, Yin D, Schaffran C, Kandel DB, Kandel ER (2011). Molecular mechanism for a gateway drug: epigenetic changes initiated by nicotine prime gene expression by cocaine. Science Translational Medicine 3, 107ra109.

Kandel ER, Kandel DB (2014). Shattuck Lecture. A molecular basis for nicotine as a gateway drug. New England Journal of Medicine 371, 932–943

Huang YY, Kandel DB, Kandel ER, Levine A (2013). Nicotine primes the effect of cocaine on the induction of LTP in the amygdala. Neuropharmacology 74, 126–134. Hughes J (2008). Smoking and suicide: a brief overview. Drug and Alcohol Dependence 98, 169–178.

Pandey GN (2013). Biological basis of suicide and suicidal behavior. Bipolar Disorders 15, 524–541.

Rom O, Avezov K, Aizenbud D, Reznick AZ (2013). Cigarette smoking and inflammation revisited. Respiratory Physiology Neurobiology 187, 5–10.

Bhang SY, Choi SW, Ahn JH (2010). Changes in plasma brain-derived neurotrophic factor levels in smokers after smoking cessation. Neuroscience Letters 468, 7–11.

Jamal M, Van der Does W, Elzinga BM, Molendijk ML, Penninx BW (2015). Association between smoking, nicotine dependence, and BDNF Val66Met polymorphism with BDNF concentrations in serum. Nicotine & Tobacco Research 17, 323–329

Zhang XY, Tan YL, Chen DC, Tan SP, Yang FD, Zunta-Soares GB, Soares JC (2016). Effects of cigarette smoking and alcohol use on neurocognition and BDNF levels in a Chinese population. Psychopharmacology (Berl) 233, 435–445.

Kirch DG, Gerhardt GA, Shelton RC, Freedman R, Wyatt RJ (1987). Effect of chronic nicotine administration on monoamine and monoamine metabolite concentrations in rat brain. Clinical Neuropharmacology 10, 376–383.

Oquendo MA, Sullivan GM, Sudol K, Baca-Garcia E, Stanley BH, Sublette ME, Mann JJ (2014). Toward a biosignature for suicide. American Journal of Psychiatry 171, 1259–1277.

© 2024 Lee PH et al. *JAMA Network Open.*

Kolokotroni KZ, Rodgers RJ, Harrison AA (2014). Trait differences in response to chronic nicotine and nicotine withdrawal in rats. Psychopharmacology (Berlin) 231, 567–580.

Kayir H, Semenova S, Markou A (2014). Baseline impulsive choice predicts the effects of nicotine and nicotine withdrawal on impulsivity in rats. Progress in Neuropsychopharmacology and Biological Psychiatry 48, 6–13.

Durazzo TC, Meyerhoff DJ, Mon A, Abe C, Gazdzinski S, Murray DE (2016). Chronic cigarette smoking in healthy middle-aged individuals is associated with decreased regional brain N-acetylaspartate and glutamate levels. Biological Psychiatry 79, 481–488.

van Heeringen K, Mann JJ (2014). The neurobiology of suicide. Lancet Psychiatry 1, 63–72.

Malone KM, Waternaux C, Haas GL, Cooper TB, Li S, Mann JJ (2003). Cigarette smoking, suicidal behavior, and serotonin function in major psychiatric disorders. American Journal of Psychiatry 160, 773–779.

© 2024 Lee PH et al. *JAMA Network Open.*

eFigure 1. Number of Study Participants who Endorsed Suicide Attempts (SAs), Ideation (SI), and Non-Suicidal Self-Injuries (NSSI) from the KSADS-5, and Case Group Classifications Used in the Present Study. To enhance our statistical power to identify unique characteristics of each SITB group, we defined NSSI, SA, and SI cases as distinct, non- overlapping categories. Specifically, participants who reported engaging in suicidal attempts (either interrupted/aborted/actual) were categorized as SA cases, those who reported experiencing suicidal ideation without attempts were categorized as SI cases, and individuals who engaged in non-suicidal self-injury without endorsing suicidal attempts or ideation were classified as NSSI cases. In each assessment, the first diagram shows the number of study participants that endorsed each SITB experiences, while the second diagram shows the number of cases classified based on our criteria.

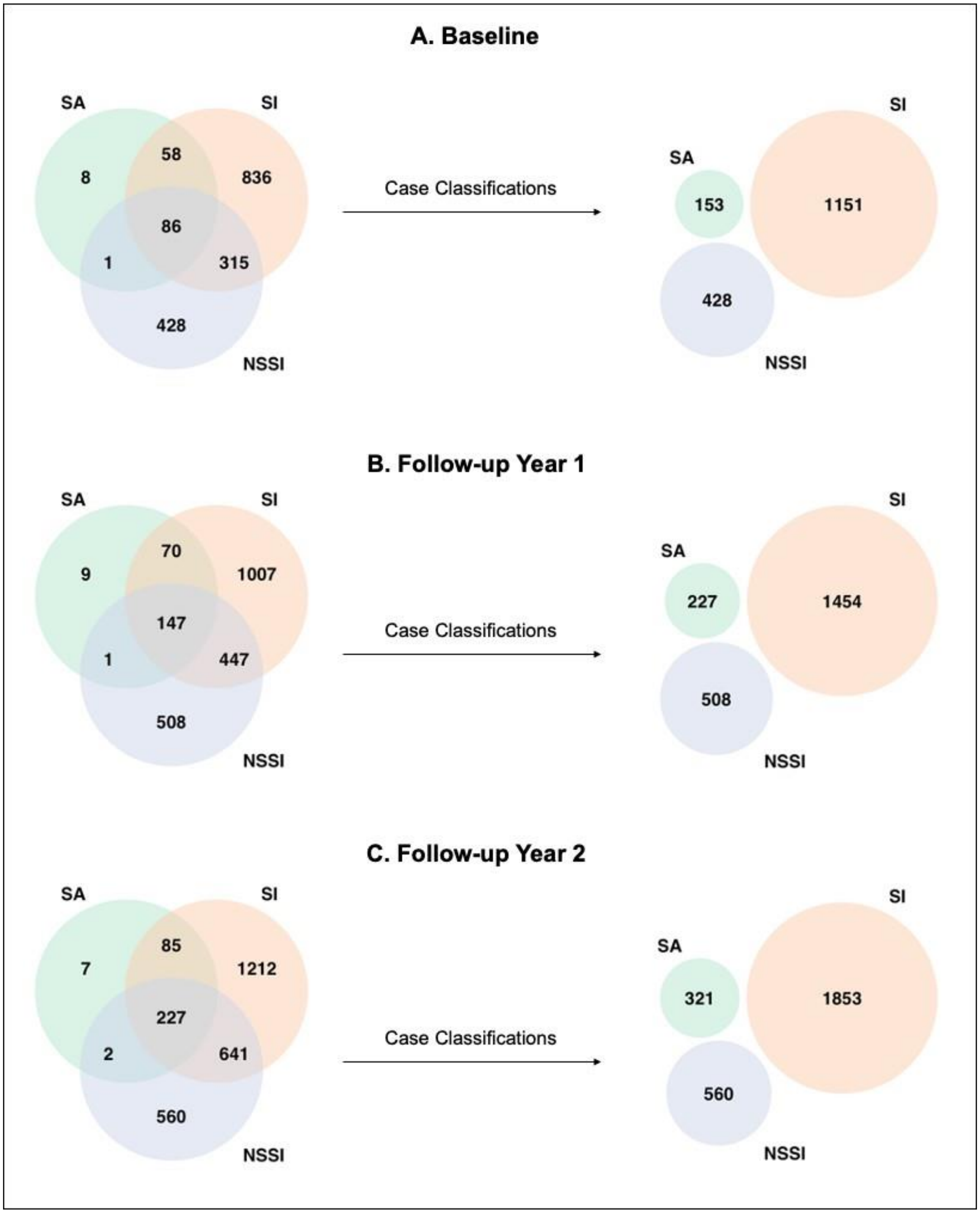

© 2024 Lee PH et al. *JAMA Network Open.*

eFigure 2. Flowchart for Study Participant Selection**.** The present study analyzed 8988 participants who had complete data of sociodemographic, family history, prenatal substance exposure, suicide risk outcomes, use of tobacco products, and children's psychopathology, temperament, and cognition measures that where significant correlates of smoking at baseline. In all follow-up analyses, listwise deletion was used for missing data**.**

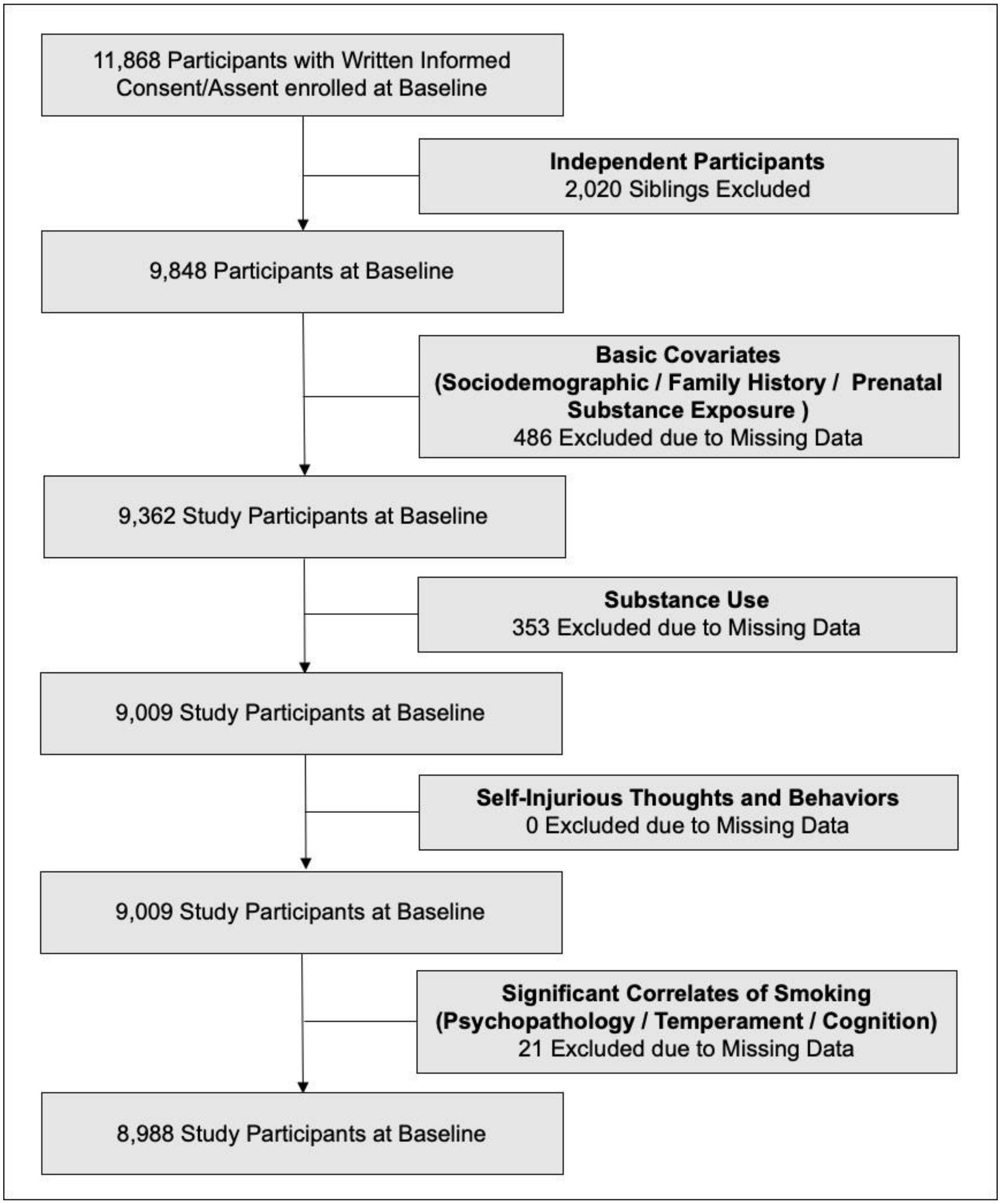

© 2024 Lee PH et al. *JAMA Network Open.*

eFigure 3. Association Between the Use of Tobacco Products (UTPs) and Suicide Risk Outcomes Based on the Basic Model[a]

Predictor	Outcome	Odds Ratio (95% CI)	FDR
Smoking (Baseline)	NSSI (Baseline)	1.07 (0.39–2.98)	9.33e–01
	NSSI (Year 1)	0.96 (0.34–2.67)	9.33e–01
	NSSI (Year 2)	1.08 (0.43–2.74)	9.33e–01
	SA (Baseline)	6.97 (3.61–13.43)	3.21e–08
	SA (Year 1)	6.58 (3.71–11.66)	9.99e–10
	SA (Year 2)	4.50 (2.55–7.92)	5.97e–07
	SI (Baseline)	2.48 (1.55–3.96)	3.86e–04
	SI (Year 1)	2.37 (1.50–3.74)	4.79e–04
	SI (Year 2)	1.91 (1.22–3.00)	8.21e–03
Smoking (6 Month)	NSSI (Year 1)	1.15 (0.49–2.66)	9.01e–01
	NSSI (Year 2)	1.19 (0.54–2.63)	8.46e–01
	SA (Year 1)	6.15 (3.60–10.50)	5.58e–10
	SA (Year 2)	4.21 (2.48–7.14)	3.78e–07
	SI (Year 1)	2.13 (1.40–3.24)	8.48e–04
	SI (Year 2)	1.69 (1.12–2.55)	1.88e–02
Smoking (18 Month)	NSSI (Year 2)	1.44 (0.74–2.81)	3.89e–01
	SA (Year 2)	4.48 (2.79–7.19)	2.72e–09
	SI (Year 2)	1.63 (1.12–2.38)	1.82e–02

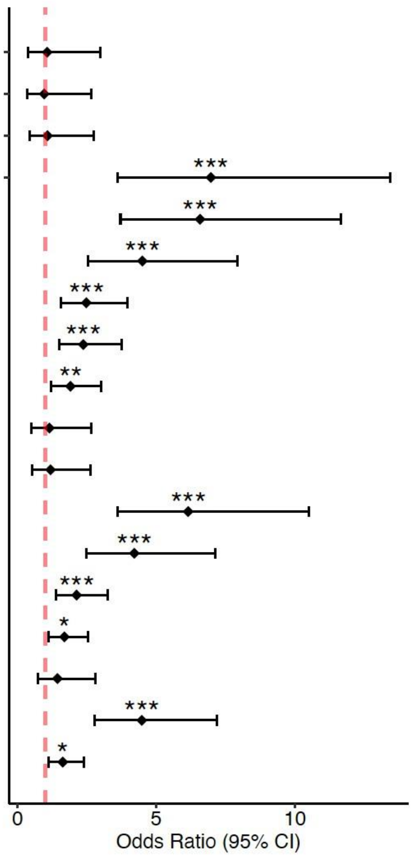

[a] Multivariate logistic regression was conducted to examine the association between UTPs and suicidal behavior outcomes, non-suicidal self-injury (NSSI), suicidal ideation (SI), and suicide attempts (SAs). Both exposure and outcome measures were binary variables. In the basic regression model, UTPs and each suicidal behavior outcome were tested as an independent predictor and a dependent variable, respectively, while controlling for demographic variables (age, sex, race).

© 2024 Lee PH et al. *JAMA Network Open.*

eTable 1. List of 22 ABCD Study Enrollment Sites

The study site information was obtained from the ABCD website: https://abcdstudy.org/study-sites/.

Study Site	Principal Investigator(s)	Contact Email	Location
Children's Hospital Los Angeles	Drs. Elizabeth Sowell and Megan Herting	abcd@chla.usc.edu	Los Angeles, CA
Florida International University	Drs. Raul Gonzalez and Angela Laird	ABCD@fiu.edu	Miami, FL
Laureate Institute for Brain Research	Drs. Martin P. Paulus and Robin Aupperle	abcd@libr.net	Tulsa, OK
Medical University of South Carolina	Drs. Lindsay Squeglia and Kevin Gray	abcdstudy@musc.edu	Charleston, SC
Oregon Health & Science University	Drs. Bonnie J. Nagel, Sarah W. Feldstein Ewing and Damien Fair	abcd@ohsu.edu	Portland, OR
SRI International	Drs. Fiona Baker and Eva Müller-Oehring	abcd@sri.com	Menlo Park, CA
UC San Diego	Drs. Susan Tapert and Joanna Jacobus	abcd@ucsd.edu	La Jolla, CA
UCLA	Drs. Susan Bookheimer and Mirella Dapretto	abcdstudy@mednet.ucla.edu	Los Angeles, CA
University of Colorado Boulder	Drs. Marie Banich and Naomi Friedman	abcd@colorado.edu	Boulder, CO
University of Florida	Drs. Sara Jo Nixon and Linda Cottler	abcd@health.ufl.edu	Gainesville, FL
University of Maryland at Baltimore	Drs. Thomas Ernst and Linda Chang	abcd@som.umaryland.edu	Baltimore, MD
University of Michigan	Drs. Mary Heitzeg and Chandra Sripada	abcdmichigan@umich.edu	Ann Arbor, MI
University of Minnesota	Drs. Monica M. Luciana and Sylia Wilson	abcd@umn.edu	Minneapolis, MN
University of Pittsburgh	Drs. Duncan Clark and Beatriz Luna	abcd@upmc.edu	Pittsburgh, PA
University of Rochester	Drs. John Foxe and Edward Freedman	ABCD@urmc.rochester.edu	Rochester, NY
University of Utah	Drs. Deborah Yurgelun-Todd and Perry Renshaw	abcd@utah.edu	Salt Lake City, UT
University of Vermont	Dr. Alexandra Potter	abcd@uvm.edu	Burlington, VT
University of Wisconsin-Milwaukee	Drs. Krista Lisdahl and Christine Larson	abcduwm@gmail.com	Wauwatosa, WI
Virginia Commonwealth University	Drs. James Bjork and Mike Neale	abcd@vcuhealth.org	Richmond, VA
Washington University in St. Louis	Drs. Pamela Madden, Deanna M. Barch and Andrew Heath	abcd@wustl.edu	St. Louis, MO
Yale University	Drs. Arielle Baskin Sommers, B.J. Casey and Dylan Gee	ABCD@yale.edu	New Haven, CT

© 2024 Lee PH et al. *JAMA Network Open.*

eTable 2. Details of Children's Tobacco Use Outcome Measures[a]

Variable	Description	ABCD Field	ABCD Value Ranges	ABCD Instrument	ABCD File
Tobacco Use (Baseline)	If child has tried puff from tobacco, e-cigarette, vape pen, or e-hookah	tlfb_tob_puff	0 = No; 1 = Yes	ABCD Youth Substance Use Interview	aqabcd_ysu02
	If child has used tobacco cigarette - more than a puff	tlfb_cig_use	0 = No; 1 = Yes		
	If child has used e-cigarettes, vape pens, or e-hookah - more than a puff	tlfb_ecig_use	0 = No; 1 = Yes		
	If child has used smokeless tobacco, "chew" or snus	tlfb_chew_use	0 = No; 1 = Yes		
	If child has used cigars, little cigars, or cigarillos	tlfb_cigar_use	0 = No; 1 = Yes		
	If child has used hookah	tlfb_hookah_use	0 = No; 1 = Yes		
	If child has used pipes	tlfb_pipes_use	0 = No; 1 = Yes		
	If child has used nicotine replacements (such as patches, gums, nasal sprays, inhalers, and lozenges)	tlfb_nicotine_use	0 = No; 1 = Yes		
	If child has used tobacco combined with marijuana	tlfb_blunt_use	0 = No; 1 = Yes		
	Presence of cotinine in subject hair sample	hair_results_cotinine_scrn	0 = NEG; 1 = POS; 2 = QNS	ABCD Youth Hair Results	abcd_yhr01

© 2024 Lee PH et al. *JAMA Network Open.*

Variable	Description	ABCD Field	ABCD Value Ranges	ABCD Instrument	ABCD File
Tobacco Use (6 & 18 Months)	If child has smoked full tobacco cigarette, cigar, pipe, multiple puffs of hookah, or an electronic cigarette in the past 6 months	mypi_tob_used	1 = yes; 0 = no; 777 = refuse to answer	ABCD Youth Mid-Year Phone Interview Substance Use	abcd_ymypisu01
	If child has smoked full tobacco cigarette, cigar, pipe, multiple puffs of hookah, or an electronic cigarette 5 or more times in the past 6 months	mypi_tob_pst_6mo	1 = yes; 0 = no; 777 = refuse to answer		
	If child has smoked full tobacco cigarette, cigar, pipe, multiple puffs of hookah, or an electronic cigarette in the past month	mypi_tob_pst_mo	1 = yes; 0 = no; 777 = refuse to answer		
	If child has smoked full tobacco cigarette, cigar, pipe, multiple puffs of hookah, or an electronic cigarette in the past week	mypi_tob_lst_wk	1 = yes; 0 = no; 777 = refuse to answer		
	If child has had a puff of a tobacco cigarette, cigar, pipe, multiple puffs of hookah, or an electronic cigarette in the past 6 months	mypi_ecig	1 = yes; 0 = no; 777 = refuse to answer		

© 2024 Lee PH et al. *JAMA Network Open.*

Variable	Description	ABCD Field	ABCD Value Ranges	ABCD Instrument	ABCD File
	If child has had a puff of a tobacco cigarette, cigar, pipe, multiple puffs of hookah, or an electronic cigarette 5 or more times in the past 6 months	mypi_ecig_pst_6mo	1 = yes; 0 = no; 777 = refuse to answer		
	If child has had a puff of a tobacco cigarette, cigar, pipe, multiple puffs of hookah, or an electronic cigarette in the past month	mypi_ecig_pst_mo	1 = yes; 0 = no; 777 = refuse to answer		
	If child has had a puff of a tobacco cigarette, cigar, pipe, multiple puffs of hookah, or an electronic cigarette in the past week	mypi_ecig_lst_wk	1 = yes; 0 = no; 777 = refuse to answer		
	If child has used smokeless tobacco, "chew", or snus in the past 6 months	mypi_chew_pst_used	1 = yes; 0 = no; 777 = refuse to answer		
	If child has used smokeless tobacco, “chew", or snus 5 or more times in the past 6 months	mypi_chew_pst_6mo	1 = yes; 0 = no; 777 = refuse to answer		
	If child has used smokeless tobacco, “chew", or snus in the past month	mypi_chew_pst_mo	1 = yes; 0 = no; 777 = refuse to answer		
	If child has used smokeless tobacco, “chew",	mypi_chew_lst_wk	1 = yes; 0 = no; 777 = refuse		

© 2024 Lee PH et al. *JAMA Network Open.*

Variable	Description	ABCD Field	ABCD Value Ranges	ABCD Instrument	ABCD File
	or snus in the past week		to answer		
	If child has smoked a tobacco cigar, hookah, or pipe in the past 6 months	mypi_full_cig	1=yes; 0=no; 777=refuse to answer [mypi_ecig] = '1' and ([event-name] = "30_month_follow_up_arm_1" or [event-name] = "42_month_follow_up_arm_1" OR [event-name] = '54_month_follow_up_arm_1')		

[a] Children's substance use data were assessed in the baseline using the ABCD Youth Substance Use Interview. The ABCD Youth Mid-Year Phone Interview for Substance Use was conducted at 6 month and 18-month follow-ups, which assessed whether they have smoked the tobacco products at any time in the past 6 months. In addition, hair toxicological screening test results were available for the baseline, year 1 and Year 2. Using these three instruments, we generated lifetime use of tobacco products variables for the baseline, 6 months, and 18-month follow-ups (binary).

© 2024 Lee PH et al. *JAMA Network Open.*

eTable 3. Details of KSADS-5 Variables used for Constructing Lifetime Suicide Risk and Self-Injury Outcome Measures[a]

Present refers to the study participant's risk experienced in the past two weeks, while Past refers to the experiences in any past time.

Outcome Measure	KSADS-5 Items	ABCD KSADS-5 Descriptions
SA	ksads_23_952	Diagnosis - Interrupted Attempt, Present
	ksads_23_953	Diagnosis - Aborted Attempt, Present
	ksads_23_954	Diagnosis - Suicide Attempt, Present
	ksads_23_963	Diagnosis - Interrupted Attempt, Past
	ksads_23_964	Diagnosis - Aborted Attempt, Past
	ksads_23_965	Diagnosis - Suicide Attempt, Past
SI	ksads_23_946	Diagnosis - Suicidal Ideation Passive, Present
	ksads_23_947	Diagnosis - Suicidal Ideation Active on Specific, Present
	ksads_23_948	Diagnosis - Suicidal Ideation Active Method, Present
	ksads_23_949	Diagnosis - Suicidal Ideation Active Intent, Present
	ksads_23_950	Diagnosis - Suicidal Ideation Active Plan, Present
	ksads_23_951	Diagnosis - Preparatory Actions Toward Imminent Suicidal Behavior, Present
	ksads_23_957	Diagnosis - Suicidal Ideation Passive, Past
	ksads_23_958	Diagnosis - Suicidal Ideation Active on Specific, Past
	ksads_23_959	Diagnosis - Suicidal Ideation Active Method, Past
	ksads_23_960	Diagnosis - Suicidal Ideation Active Intent, Past
	ksads_23_961	Diagnosis - Suicidal Ideation Active Plan, Past
	ksads_23_962	Diagnosis - Preparatory Actions Toward Imminent Suicidal Behavior, Past
NSSI	ksads_23_945	Diagnosis - Self Injurious Behavior Without Suicidal Intent, Present
	ksads_23_956	Diagnosis - Self Injurious Behavior Without Suicidal Intent, Past

[a] SA: suicide attempts, SI: suicidal ideation; NSSI: non-suicidal self-injuries

© 2024 Lee PH et al. *JAMA Network Open.*

eTable 4. Response Rates for UTP and SITB Measures

In the '# of All subjects' column, you can find the number of study participants with available data in ABCD data release v4 for each variable. The '# of valid responses' column indicates the number of participants who answered either 'yes' or 'no' to the questions. Response rates (%) are calculated by dividing the number of participants with valid responses by the total number of individuals surveyed in each assessment. The 'Yes Response Rates (%)' column shows the rates of participants who answered 'yes' among those with valid responses. For detailed information about the ABCD variables summarized in this table, including instrument descriptions, please refer to eTables 2 and 3.

Assessment	ABCD Variable	# All Subjects	# Valid Responses	Response Rates (%)	# Yes Responses	# of No Responses	Yes Response Rates (%)
Baseline	tlfb_tob_puff	11876	11083	93.32	81	11002	0.73
Baseline	tlfb_cig_use	11876	81	0.68	9	72	11.11
Baseline	tlfb_ecig_use	11876	81	0.68	11	70	13.58
Baseline	tlfb_chew_use	11876	11086	93.35	12	11074	0.11
Baseline	tlfb_cigar_use	11876	11083	93.32	10	11073	0.09
Baseline	tlfb_hookah_use	11876	11083	93.32	7	11076	0.06
Baseline	tlfb_pipes_use	11876	11083	93.32	5	11078	0.05
Baseline	tlfb_nicotine_use	11876	11083	93.32	8	11075	0.07
Baseline	tlfb_blunt_use	11876	12	0.1	1	11	8.33
Baseline	cotinine	445	445	100	26	419	5.84
Year 1	cotinine	357	357	100	4	353	1.12
Year 2	cotinine	463	463	100	1	462	0.22
6 Month	mypi_tob_used	11396	9868	86.59	14	9854	0.14
6 Month	mypi_tob_pst_6mo	11396	14	0.12	1	13	7.14
6 Month	mypi_tob_pst_mo	11396	14	0.12	4	10	28.57
6 Month	mypi_tob_lst_wk	11396	4	0.04	3	1	75
6 Month	mypi_ecig	11396	9854	86.47	21	9833	0.21
6 Month	mypi_ecig_pst_6mo	11396	21	0.18	1	20	4.76
6 Month	mypi_ecig_pst_mo	11396	21	0.18	3	18	14.29
6 Month	mypi_ecig_lst_wk	11396	3	0.03	1	2	33.33
6 Month	mypi_chew_pst_used	11396	4665	40.94	7	4658	0.15
6 Month	mypi_chew_pst_6mo	11396	7	0.06	0	7	0
6 Month	mypi_chew_pst_mo	11396	7	0.06	3	4	42.86
6 Month	mypi_chew_lst_wk	11396	3	0.03	2	1	66.67
18 Month	mypi_tob_used	11089	9972	89.93	11	9961	0.11

© 2024 Lee PH et al. *JAMA Network Open.*

18 Month	mypi_tob_pst_6mo	11089	11	0.1	2	9	18.18
18 Month	mypi_tob_pst_mo	11089	11	0.1	2	9	18.18
18 Month	mypi_tob_lst_wk	11089	2	0.02	0	2	0
18 Month	mypi_ecig	11089	9959	89.81	25	9934	0.25
18 Month	mypi_ecig_pst_6mo	11089	25	0.23	1	24	4
18 Month	mypi_ecig_pst_mo	11089	25	0.23	5	20	20
18 Month	mypi_ecig_lst_wk	11089	5	0.05	2	3	40
18 Month	mypi_chew_pst_used	11089	5811	52.4	3	5808	0.05
18 Month	mypi_chew_pst_6mo	11089	3	0.03	0	3	0
18 Month	mypi_chew_pst_mo	11089	3	0.03	0	3	0
18 Month	mypi_chew_lst_wk	11089	0	0	0	0	0
Baseline	ksads_23_952_y	11876	11804	99.39	6	11798	0.05
Baseline	ksads_23_953_y	11876	11804	99.39	20	11784	0.17
Baseline	ksads_23_954_y	11876	11798	99.34	23	11775	0.19
Baseline	ksads_23_963_y	11876	11804	99.39	11	11793	0.09
Baseline	ksads_23_964_y	11876	11804	99.39	36	11768	0.3
Baseline	ksads_23_965_y	11876	11804	99.39	86	11718	0.73
Baseline	ksads_23_946_y	11876	11804	99.39	142	11662	1.2
Baseline	ksads_23_947_y	11876	11804	99.39	191	11613	1.62
Baseline	ksads_23_948_y	11876	11804	99.39	63	11741	0.53
Baseline	ksads_23_949_y	11876	11804	99.39	37	11767	0.31
Baseline	ksads_23_950_y	11876	11804	99.39	32	11772	0.27
Baseline	ksads_23_951_y	11876	11804	99.39	24	11780	0.2
Baseline	ksads_23_957_y	11876	11804	99.39	703	11101	5.96
Baseline	ksads_23_958_y	11876	11804	99.39	430	11374	3.64
Baseline	ksads_23_959_y	11876	11804	99.39	108	11696	0.91
Baseline	ksads_23_960_y	11876	11804	99.39	60	11744	0.51
Baseline	ksads_23_961_y	11876	11804	99.39	37	11767	0.31
Baseline	ksads_23_962_y	11876	11804	99.39	41	11763	0.35
Baseline	ksads_23_945_y	11876	11804	99.39	319	11485	2.7
Baseline	ksads_23_956_y	11876	11804	99.39	507	11297	4.3
Year 1	ksads_23_952_y	11225	11100	98.89	7	11093	0.06
Year 1	ksads_23_953_y	11225	11100	98.89	13	11087	0.12
Year 1	ksads_23_954_y	11225	11100	98.89	12	11088	0.11
Year 1	ksads_23_963_y	11225	11100	98.89	14	11086	0.13
Year 1	ksads_23_964_y	11225	11100	98.89	44	11056	0.4

© 2024 Lee PH et al. *JAMA Network Open.*

Year 1	ksads_23_965_y	11225	11100	98.89	83	11017	0.75
Year 1	ksads_23_946_y	11225	11100	98.89	111	10989	1
Year 1	ksads_23_947_y	11225	11100	98.89	144	10956	1.3
Year 1	ksads_23_948_y	11225	11100	98.89	47	11053	0.42
Year 1	ksads_23_949_y	11225	11100	98.89	39	11061	0.35
Year 1	ksads_23_950_y	11225	11100	98.89	22	11078	0.2
Year 1	ksads_23_951_y	11225	11100	98.89	22	11078	0.2
Year 1	ksads_23_957_y	11225	11100	98.89	683	10417	6.15
Year 1	ksads_23_958_y	11225	11100	98.89	427	10673	3.85
Year 1	ksads_23_959_y	11225	11100	98.89	116	10984	1.05
Year 1	ksads_23_960_y	11225	11100	98.89	84	11016	0.76
Year 1	ksads_23_961_y	11225	11100	98.89	35	11065	0.32
Year 1	ksads_23_962_y	11225	11100	98.89	44	11056	0.4
Year 1	ksads_23_945_y	11225	11100	98.89	192	10908	1.73
Year 1	ksads_23_956_y	11225	11100	98.89	418	10682	3.77
Year 2	ksads_23_952_y	10414	10313	99.03	4	10309	0.04
Year 2	ksads_23_953_y	10414	10313	99.03	11	10302	0.11
Year 2	ksads_23_954_y	10414	10311	99.01	12	10299	0.12
Year 2	ksads_23_963_y	10414	10313	99.03	8	10305	0.08
Year 2	ksads_23_964_y	10414	10313	99.03	26	10287	0.25
Year 2	ksads_23_965_y	10414	10313	99.03	111	10202	1.08
Year 2	ksads_23_946_y	10414	10313	99.03	95	10218	0.92
Year 2	ksads_23_947_y	10414	10313	99.03	133	10180	1.29
Year 2	ksads_23_948_y	10414	10313	99.03	49	10264	0.48
Year 2	ksads_23_949_y	10414	10313	99.03	26	10287	0.25
Year 2	ksads_23_950_y	10414	10313	99.03	24	10289	0.23
Year 2	ksads_23_951_y	10414	10313	99.03	17	10296	0.16
Year 2	ksads_23_957_y	10414	10313	99.03	625	9688	6.06
Year 2	ksads_23_958_y	10414	10313	99.03	380	9933	3.68
Year 2	ksads_23_959_y	10414	10313	99.03	101	10212	0.98
Year 2	ksads_23_960_y	10414	10313	99.03	87	10226	0.84
Year 2	ksads_23_961_y	10414	10313	99.03	33	10280	0.32
Year 2	ksads_23_962_y	10414	10313	99.03	38	10275	0.37
Year 2	ksads_23_945_y	10414	10313	99.03	145	10168	1.41
Year 2	ksads_23_956_y	10414	10313	99.03	364	9949	3.53
Baseline	ksads_23_952_p	11876	11735	98.81	0	11735	0
Baseline	ksads_23_953_p	11876	11735	98.81	1	11734	0.01
Baseline	ksads_23_954_p	11876	11735	98.81	7	11728	0.06
Baseline	ksads_23_963_p	11876	11735	98.81	7	11728	0.06

© 2024 Lee PH et al. *JAMA Network Open.*

Baseline	ksads_23_964_p	11876	11735	98.81	9	11726	0.08
Baseline	ksads_23_965_p	11876	11735	98.81	32	11703	0.27
Baseline	ksads_23_946_p	11876	11735	98.81	63	11672	0.54
Baseline	ksads_23_947_p	11876	11735	98.81	132	11603	1.12
Baseline	ksads_23_948_p	11876	11735	98.81	20	11715	0.17
Baseline	ksads_23_949_p	11876	11735	98.81	9	11726	0.08
Baseline	ksads_23_950_p	11876	11735	98.81	4	11731	0.03
Baseline	ksads_23_951_p	11876	11735	98.81	12	11723	0.1
Baseline	ksads_23_957_p	11876	11735	98.81	759	10976	6.47
Baseline	ksads_23_958_p	11876	11735	98.81	370	11365	3.15
Baseline	ksads_23_959_p	11876	11735	98.81	98	11637	0.84
Baseline	ksads_23_960_p	11876	11735	98.81	43	11692	0.37
Baseline	ksads_23_961_p	11876	11735	98.81	16	11719	0.14
Baseline	ksads_23_962_p	11876	11735	98.81	51	11684	0.43
Baseline	ksads_23_945_p	11876	11735	98.81	182	11553	1.55
Baseline	ksads_23_956_p	11876	11735	98.81	392	11343	3.34
Year 2	ksads_23_952_p	10414	10216	98.1	1	10215	0.01
Year 2	ksads_23_953_p	10414	10216	98.1	2	10214	0.02
Year 2	ksads_23_954_p	10414	10215	98.09	9	10206	0.09
Year 2	ksads_23_963_p	10414	10216	98.1	8	10208	0.08
Year 2	ksads_23_964_p	10414	10216	98.1	6	10210	0.06
Year 2	ksads_23_965_p	10414	10216	98.1	62	10154	0.61
Year 2	ksads_23_946_p	10414	10216	98.1	64	10152	0.63
Year 2	ksads_23_947_p	10414	10216	98.1	129	10087	1.26
Year 2	ksads_23_948_p	10414	10216	98.1	24	10192	0.23
Year 2	ksads_23_949_p	10414	10216	98.1	9	10207	0.09
Year 2	ksads_23_950_p	10414	10216	98.1	7	10209	0.07
Year 2	ksads_23_951_p	10414	10216	98.1	13	10203	0.13
Year 2	ksads_23_957_p	10414	10216	98.1	704	9512	6.89
Year 2	ksads_23_958_p	10414	10216	98.1	514	9702	5.03
Year 2	ksads_23_959_p	10414	10216	98.1	135	10081	1.32
Year 2	ksads_23_960_p	10414	10216	98.1	61	10155	0.6
Year 2	ksads_23_961_p	10414	10216	98.1	29	10187	0.28
Year 2	ksads_23_962_p	10414	10216	98.1	65	10151	0.64
Year 2	ksads_23_945_p	10414	10216	98.1	209	10007	2.05
Year 2	ksads_23_956_p	10414	10216	98.1	390	9826	3.82

© 2024 Lee PH et al. *JAMA Network Open.*

eTable 5. Data on Sociodemographics, Family History, and Prenatal Exposure to Substance Use

Category	Variable	ABCD Field	ABCD Survey	File Name
Demographics	Age	interview_age	Parent Demographics Survey	pdem02
	Sex	sex		
	Race/Ethnicity	race_ethnicity		
Socioeconomic Status	Parental Education Level	demo_prnt_ed_v2		
		demo_prtnr_ed_v2		
	Household Income Level	demo_comb_income_v2		
	Poverty Status			
	Parent Marital Status	demo_prnt_marital_v2		
Family History	Parental History of Suicide	famhx_ss_fath_prob_scd_p	Parent Family History Summary Scores	abcd_fhxss p01
		famhx_ss_moth_prob_scd_p		
	Parental History of Depression	famhx_ss_fath_prob_dprs_p		
		famhx_ss_moth_prob_dprs_p		
	Parental History of Alcohol Use Problem	famhx_ss_fath_prob_alc_p		
		famhx_ss_moth_prob_alc_p		
	Parental History of Drug Use Problem	famhx_ss_fath_prob_dg_p		
		famhx_ss_moth_prob_dg_p		
	Parental History of Mania	famhx_ss_fath_prob_ma_p		
		famhx_ss_moth_prob_ma_p		
	Parental History of Nerves/Nervous Breakdown Problems	famhx_ss_fath_prob_nrv_p		
		famhx_ss_moth_prob_nrv_p		
	Parental History of Troubles Holding Jobs/Fights/Police Problems	famhx_ss_fath_prob_trb_p		
		famhx_ss_moth_prob_trb_p		
	Parental History of Hospitalization due to Emotional/Mental Problems	famhx_ss_fath_prob_hspd_p		
		famhx_ss_moth_prob_hspd_p		
	Parental History of seeing Doctors for Emotional/Mental Problems	famhx_ss_fath_prob_prf_p		
		famhx_ss_moth_prob_prf_p		

© 2024 Lee PH et al. *JAMA Network Open.*

Category	Variable	ABCD Field	ABCD Survey	File Name
Early Prenatal Exposure	Maternal use of tobacco prior to knowledge of pregnancy	devhx_8_tobacco	ABCD Developmental History Questionnaire	dhx01
	Maternal use of cocaine/crack prior to knowledge of pregnancy	devhx_8_coc_crack		
	Maternal use of heroin/morphine prior to knowledge of pregnancy	devhx_8_her_morph		
	Maternal use of oxycontin prior to knowledge of pregnancy	devhx_8_oxycont		
	Maternal use of any other drugs prior to knowledge of pregnancy	devhx_8_other_drugs		
Late Prenatal Exposure	Maternal use of tobacco after to knowledge of pregnancy	devhx_9_tobacco		
	Maternal use of cocaine/crack after to knowledge of pregnancy	devhx_9_coc_crack		
	Maternal use of heroin/morphine after to knowledge of pregnancy	devhx_9_her_morph		
	Maternal use of oxycontin after to knowledge of pregnancy	devhx_9_oxycont		
	Maternal use of any other drugs after to knowledge of pregnancy	devhx_9_other_drugs		

© 2024 Lee PH et al. *JAMA Network Open.*

eTable 6. Child Behavioral Outcome Measures

Category	Variable	ABCD Field	ABCD Survey	File Name
Child Psychopathology	Anxious/Depressed	cbcl_scr_syn_anxdep_t	Parent Child Behavior Checklist Scores Aseba (CBCL)	abcd_cbcls01
	Withdrawn/Depressed	cbcl_scr_syn_withdep_t		
	Somatic Complaints	cbcl_scr_syn_somatic_t		
	Social Problems	cbcl_scr_syn_social_t		
	Thought Problems	cbcl_scr_syn_thought_t		
	Attention Problems	cbcl_scr_syn_attention_t		
	Rule-Breaking Behavior	cbcl_scr_syn_rulebreak_t		
	Aggressive Behavior	cbcl_scr_syn_aggressive_t		
	Internalizing Problems	cbcl_scr_syn_internal_t		
	Externalizing Problems	cbcl_scr_syn_external_t		
	Total Problems	cbcl_scr_syn_totprob_t		
	Depressive Disorder	cbcl_scr_dsm5_depress_t		
	Anxiety Disorder	cbcl_scr_dsm5_anxdisord_t		
	ADHD	cbcl_scr_dsm5_adhd_t		
	Oppositional Defiant Disorder	cbcl_scr_dsm5_opposit_t		
	Conduct Disorder	cbcl_scr_dsm5_conduct_t		
Cognition	Vocabulary	nihtbx_picvocab_agecorrected	Youth NIH TB Summary Scores	abcd_tbss01
	Processing Speed	nihtbx_pattern_agecorrected		
	Attention	nihtbx_flanker_agecorrected		
	Executive Function	nihtbx_cardsort_agecorrected		
	Crystallized Cognition	nihtbx_cryst_agecorrected		
	Episodic Memory	nihtbx_picture_agecorrected		
	Reading	nihtbx_reading_agecorrected		
	Short Delay Recall	pea_ravlt_sd_trial_vi_tc		
	Long Delay Recall	pea_ravlt_ld_trial_vii_tc		
	Visuospatial Accuracy	lmt_scr_perc_correct		
	Visuospatial Reaction Time	lmt_scr_rt_correct		
Personality / Temperament	Activation Control	eatq_finish_p, eatq_deal_p, eatq_before_hw_p, eatq_right_away_p, eatq_finish_hw_p,	abcd_eatqp 01	abcd_eatqp01

© 2024 Lee PH et al. *JAMA Network Open.*

Category	Variable	ABCD Field	ABCD Survey	File Name
		eatq_early_start_p eatq_puts_off_p		
	Affiliation	eatq_care_p, eatq_share_p, eatq_spend_time_p eatq_hugs_p, eatq_close_rel_p, eatq_friendly_p		
	Aggression	eatq_insult_p, eatq_angry_hit_p, eatq_rude_p, eatq_blame_p, eatq_doorslam_p, eatq_makes_fun_p, eatq_no_criticize_p		
	Attention	eatq_concentrate_p, eatq_distracted_p, eatq_try_focus_p, eatq_peripheral_p, eatq_sidetracked_p, eatq_close_attention_p		
	Depressive Mood	eatq_enjoy_p, eatq_cry_p, eatq_sad_p, eatq_hardly_sad_p eatq_seems_sad_p		
	Fear	eatq_trouble_p, eatq_worry_p, eatq_attachment_p, eatq_ball_scared_p eatq_dark_scared_p eatq_alone_p,		
	Frustration	eatq_annoyed_p, eatq_irritated_crit_p, eatq_irritated_place_p eatq_irritated_enjoy_p eatq_disagree_p, eatq_frustrated_p		
	High-Intensity Pleasure/Surgency	eatq_africa_p, eatq_ski_slope_p, eatq_city_move_p, eatq_sea_dive_p, eatq_travel_p, eatq_race_car_p, eatq_s		
	Inhibitory Control	eatq_turn_taking_p, eatq_open_present_p, eatq_impulse_p, eatq_laugh_control_p eatq_stick_to_plan_p		

© 2024 Lee PH et al. *JAMA Network Open.*

Category	Variable	ABCD Field	ABCD Survey	File Name
	Shyness	eatq_social_p, eatq_is_shy_p, eatq_not_shy_p, eatq_meet_p, eatq_shy_meet_p		

© 2024 Lee PH et al. *JAMA Network Open.*

eTable 7. Details of Children's Substance Use Measures[a]

Variable	Description	ABCD field	ABCD Value Ranges	ABCD Instrument	ABCD file
Alcohol Use (Sipping)	If child has ever tried a sip of alcohol such as beer, wine, or liquor (rum, vodka, gin, whiskey)	tlfb_alc_sip	0 = No; 1 = Yes	ABCD Youth Substance Use Interview	abcd_ysu02
	If child has had sip of drink containing alcohol in past 6 months	mypi_alc_sip	1 = yes; 0 = no; 777 = refuse to answer	ABCD Youth Mid-Year Phone Interview Substance Use	abcd_ymypisu01
	If child had sip of drink containing alcohol 5 or more times in the past 6 months	mypi_alc_sip_use	1 = yes; 0 = no; 777 = refuse to answer		
	If child has sip of a drink containing alcohol in the past month	mypi_alc_sip_pst_mo	1 = yes; 0 = no; 777 = refuse to answer		
	If child has had sip of a drink containing alcohol in the past week	mypi_alc_sip_lst_wk	1 = yes; 0 = no; 777 = refuse to answer		
Alcohol Use (Full Drink)	If child has had full drink of alcoholic beverage	tlfb_alc_use	0 = No; 1 = Yes	ABCD Youth Substance Use Interview	abcd_ysu02
	If child has consumed alcohol regularly	tlfb_alc_reg	0 = No; 1 = Yes		
	If child has had full drink containing alcohol in the past 6 months	mypi_alc_full_drink	1 = yes; 0 = no; 777 = refuse to answer	ABCD Youth Mid-Year Phone Interview Substance Use	abcd_ymypisu01
	If child has had full drink containing alcohol 5 or more times in the past 6 months	mypi_alc_drink_used	1 = yes; 0 = no; 777 = refuse to answer		
	If child has had full drink containing alcohol in the past month	mypi_alc_drink_pst_mo	1 = yes; 0 = no; 777 = refuse to answer		
	If child has had full drink containing alcohol in the past week	mypi_alc_drink_lst_wk	1 = yes; 0 = no; 777 = refuse to answer		
	Presence of EtG residuals in subject hair sample	hair_results_etg_scrn	0 = NEG; 1 = POS; 2 = QNS	ABCD Youth Hair Results	abcd_yhr01

© 2024 Lee PH et al. *JAMA Network Open.*

Variable	Description	ABCD field	ABCD Value Ranges	ABCD Instrument	ABCD file
Marijuana Use	If child has ever tried a puff or eaten any marijuana	tlfb_mj_puff	0 = No; 1 = Yes	ABCD Youth Substance Use Interview	abcd_ysu02
	If child has ever smoked any marijuana - more than a puff	tlfb_mj_use	0 = No; 1 = Yes		
	If child has used tobacco combined with marijuana	tlfb_blunt_use	0 = No; 1 = Yes		
	If child has ever eaten marijuana (pot cookies, gummy bears, brownies, etc.)	tlfb_edible_use	0 = No; 1 = Yes		
	If child has ever tried marijuana oils or concentrates (such as "710"; hash oil; BHO/butane hash oil/dabs/shatter/budder/honey oil; Co2 oil/vaporizer pen, etc.	tlfb_mj_conc_use	0 = No; 1 = Yes		
	If child has ever tried marijuana infused alcohol drinks	tlfb_mj_drink_use	0 = No; 1 = Yes		
	If child has ever tried concentrated marijuana tinctures	tlfb_tincture_use	0 = No; 1 = Yes		
	If child has smoked marijuana flower or bud in a pipe, joint, hookah, or bong in the past 6 months	mypi_mj_used	1 = yes; 0 = no; 777 = refuse to answer	ABCD Youth Mid-Year Phone Interview Substance Use	abcd_ymypisu01
	If child has smoked marijuana flower or bud in a pipe, joint, hookah, or bong 5 or more times in the past 6 months	mypi_mj_pst_6mo	1 = yes; 0 = no; 777 = refuse to answer		
	If child has smoked marijuana flower or bud in a pipe, joint, hookah, or bong in the past month	mypi_mj_pst_mo	1 = yes; 0 = no; 777 = refuse to answer		
	If child has smoked marijuana flower or bud in a pipe, joint, hookah, or bong in the past week	mypi_mj_lst_wk	1 = yes; 0 = no; 777 = refuse to answer		

© 2024 Lee PH et al. *JAMA Network Open.*

Variable	Description	ABCD field	ABCD Value Ranges	ABCD Instrument	ABCD file
	If child has eaten marijuana in any food (such as cookies, gummy bears, or brownies) in the past 6 months	mypi_mj_edible	1 = yes; 0 = no; 777 = refuse to answer		
	If child has eaten marijuana in any food (such as cookies, gummy bears, or brownies) 5 or more times in the past 6 months	mypi_mj_edible_pst_6mo	1 = yes; 0 = no; 777 = refuse to answer		
	If child has eaten marijuana in any food (such as cookies, gummy bears, or brownies) in the past month	mypi_mj_edible_pst_mo	1 = yes; 0 = no; 777 = refuse to answer		
	If child has eaten marijuana in any food (such as cookies, gummy bears, or brownies) in the past week	mypi_mj_edible_lst_wk	1 = yes; 0 = no; 777 = refuse to answer		
	If child has used marijuana oils or concentrates (such as hash oil, BHO, butane, or dabs) in the past 6 months	mypi_mj_oils	1 = yes; 0 = no; 777 = refuse to answer		
	If child has used marijuana oils or concentrates (such as hash oil, BHO, butane, or dabs) 5 or more times in the past 6 months	mypi_mj_oils_pst_6mo	1 = yes; 0 = no; 777 = refuse to answer		
	If child has used marijuana oils or concentrates (such as hash oil, BHO, butane, or dabs) in the past month	mypi_mj_oils_pst_mo	1 = yes; 0 = no; 777 = refuse to answer		
	If child has used marijuana oils or concentrates (such as hash oil, BHO, butane, or dabs) in the past week	mypi_mj_oils_lst_wk	1 = yes; 0 = no; 777 = refuse to answer		

© 2024 Lee PH et al. *JAMA Network Open.*

Variable	Description	ABCD field	ABCD Value Ranges	ABCD Instrument	ABCD file
	If child has had a marijuana infused alcohol drink or marijuana tincture in the past 6 months	mypi_mj_tinc_used	1 = yes; 0 = no; 777 = refuse to answer		
	If child has had a marijuana infused alcohol drink or marijuana tincture 5 or more times in the past 6 months	mypi_mj_tinc_pst_6mo	1 = yes; 0 = no; 777 = refuse to answer		
	If child has had a marijuana infused alcohol drink or marijuana tincture in the past month	mypi_mj_tinc_pst_mo	1 = yes; 0 = no; 777 = refuse to answer		
	If child has had a marijuana infused alcohol drink or marijuana tincture in the past week	mypi_mj_tinc_lst_wk	1 = yes; 0 = no; 777 = refuse to answer		
	If child has had "fake" marijuana or synthetics (such as K2 or spice) in the past 6 months	mypi_mj_synth_used	1 = yes; 0 = no; 777 = refuse to answer		
	If child has had "fake" marijuana or synthetics (such as K2 or spice) 5 or more times in the past 6 months	mypi_mj_synth_pst_6mo	1 = yes; 0 = no; 777 = refuse to answer		
	If child has had "fake" marijuana or synthetics (such as K2 or spice) in the past month	mypi_mj_synth_pst_mo	1 = yes; 0 = no; 777 = refuse to answer		
	If child has had "fake" marijuana or synthetics (such as K2 or spice) in the past week	mypi_mj_synth_lst_wk	1 = yes; 0 = no; 777 = refuse to answer		

© 2024 Lee PH et al. *JAMA Network Open.*

Variable	Description	ABCD field	ABCD Value Ranges	ABCD Instrument	ABCD file
	If child has vaped marijuana oils or concentrates (such as THC, hash, or BHO oil) in a vape pen or vaporizer in the past 6 months	mypi_mj_oils_vaped	1=yes; 0=no; 777=refuse to answer [mypi_mj] = '1' and ([event-name] = 18_month_follow_up_arm_1" or [event-name] = "6_month_follow_up_arm_1") or [mypi_mj_30] = '1' OR [event-name] = '54_month_follow_up_arm_1'"		
	If child has vaped marijuana flower or bud in a vape pen, vaporizer, or e-vaporizer any time in the past 6 months	mypi_mj_vape	1=yes; 0=no; 777=refuse to answer [mypi_mj_30] = '1' and ([event-name] = '30_month_follow_up_arm_1' or [event-name] = '42_month_follow_up_arm_1' OR [event-name] = '54_month_follow_up_arm_1')		
	Presence of Carboxy-THC residuals in subject hair sample	hair_results_thccooh_confirm	0 = NEG; 1 = POS; 2 = QNS	ABCD Youth Hair Results	abcd_yhr01

© 2024 Lee PH et al. *JAMA Network Open.*

Variable	Description	ABCD field	ABCD Value Ranges	ABCD Instrument	ABCD file
Prescription Drug Use	If child has ever tried prescription anxiolytics, tranquilizers, or sedatives (such as Xanax, Ativan, Valium, Rohypnol, or sleeping pills)	tlfb_tranq_use	0 = No; 1 = Yes	ABCD Youth Substance Use Interview	abcd_ysu02
	If child has ever tried prescription pain relievers (such as Vicodin, Lortab, Norco, Hydrocodone, Oxycontin, Percocet)	tlfb_vicodin_use	0 = No; 1 = Yes		
	If child has ever tried stimulant drugs (such as amphetamine, Ritalin, Adderall, ephedrine) in a way doctor did not direct use of	tlfb_amp_use	0 = No; 1 = Yes		
	If child has used prescription stimulant drugs (such as amphetamine, Ritalin, Adderall, or ephedrine) in the past 6 months	mypi_pills_used	1 = yes; 0 = no; 777 = refuse to answer	ABCD Youth Mid-Year Phone Interview Substance Use	abcd_ymypisu01
	If child has used prescription stimulant drugs (such as amphetamine, Ritalin, Adderall, or ephedrine) 5 or more times in the past 6 months	mypi_pills_pst_6mo	1 = yes; 0 = no; 777 = refuse to answer		
	If child has used prescription stimulant drugs (such as amphetamine, Ritalin, Adderall, or ephedrine) in the past month	mypi_pills_pst_mo	1 = yes; 0 = no; 777 = refuse to answer		
	If child has used prescription stimulant drugs (such as amphetamine, Ritalin, Adderall, or ephedrine) in the past week	mypi_pills_lst_wk	1 = yes; 0 = no; 777 = refuse to answer		

© 2024 Lee PH et al. *JAMA Network Open.*

Variable	Description	ABCD field	ABCD Value Ranges	ABCD Instrument	ABCD file
	If child has used prescription anxiolytics, tranquilizers, or sedatives to get high (such as Xanax, Ativan, Valium, Rohypnol, or sleeping pills) in the past 6 months	mypi_pills_dep_used	1 = yes; 0 = no; 777 = refuse to answer		
	If child has used prescription anxiolytics, tranquilizers, or sedatives to get high (such as Xanax, Ativan, Valium, Rohypnol, or sleeping pills) 5 or more times in the past 6 months	mypi_pills_dep_pst_6mo	1 = yes; 0 = no; 777 = refuse to answer		
	If child has used prescription anxiolytics, tranquilizers, or sedatives to get high (such as Xanax, Ativan, Valium, Rohypnol, or sleeping pills) in the past month	mypi_pills_dep_pst_mo	1 = yes; 0 = no; 777 = refuse to answer		
	If child has used prescription anxiolytics, tranquilizers, or sedatives to get high (such as Xanax, Ativan, Valium, Rohypnol, or sleeping pills) in the past week	mypi_pills_dep_lst_wk	1 = yes; 0 = no; 777 = refuse to answer		
	If child has used prescription pain relievers to get high (such as Vicodin, Lortab, Norco, Hydrocodone, Oxycontin, Percocet) in the past 6 months	mypi_pr_used	1 = yes; 0 = no; 777 = refuse to answer		

© 2024 Lee PH et al. *JAMA Network Open.*

Variable	Description	ABCD field	ABCD Value Ranges	ABCD Instrument	ABCD file
	If child has used prescription pain relievers to get high (such as Vicodin, Lortab, Norco, Hydrocodone, Oxycontin, Percocet) 5 or more times in the past 6 months	mypi_pr_pst_6mo	1 = yes; 0 = no; 777 = refuse to answer		
	If child has used prescription pain relievers to get high (such as Vicodin, Lortab, Norco, Hydrocodone, Oxycontin, Percocet) in the past month	mypi_pr_pst_mo	1 = yes; 0 = no; 777 = refuse to answer		
	If child has used prescription pain relievers to get high (such as Vicodin, Lortab, Norco, Hydrocodone, Oxycontin, Percocet) in the past week	mypi_pr_lst_wk	1 = yes; 0 = no; 777 = refuse to answer		
Over the Counter / Legal Drug and Substance Use	If child has ever tried over the counter cough or cold medicine or DXM to get high	tlfb_cough_use	0 = No; 1 = Yes	ABCD Youth Substance Use Interview	abcd_ysu02
	If child has ever tried sniffing liquids, sprays, or other products to get high	tlfb_sniff_use	0 = No; 1 = Yes		
	If child has ever tried sniffing liquids, sprays, and gases to get high (this includes poppers, correction fluid, gasoline, glue, shoe polish, spray paints, nitrous oxide of 'whippits')	tlfb_inhalant_use	0 = No; 1 = Yes		
	Which of these has child used? Check all that apply. (Poppers)	tlfb_inhalant_use_type___1	0 = No; 1 = Yes		
	Which of these has child used? Check all that apply. (Correction Fluid)	tlfb_inhalant_use_type___2	0 = No; 1 = Yes		

© 2024 Lee PH et al. *JAMA Network Open.*

Variable	Description	ABCD field	ABCD Value Ranges	ABCD Instrument	ABCD file
	Which of these has child used? Check all that apply. (Gasoline)	tlfb_inhalant_use_type___3	0 = No; 1 = Yes		
	Which of these has child used? Check all that apply. (Glue)	tlfb_inhalant_use_type___4	0 = No; 1 = Yes		
	Which of these has child used? Check all that apply. (Shoe Polish)	tlfb_inhalant_use_type___5	0 = No; 1 = Yes		
	Which of these has child used? Check all that apply. (Spray Paint)	tlfb_inhalant_use_type___6	0 = No; 1 = Yes		
	Which of these has child used? Check all that apply. (Nitrous Oxide or Whippits)	tlfb_inhalant_use_type___7	0 = No; 1 = Yes		
	Which of these has child used? Check all that apply. (Other)	tlfb_inhalant_use_type___8	0 = No; 1 = Yes		
	If child has used over the counter cough or cold medicine or DXM to get high	mypi_cold_used	1 = yes; 0 = no; 777 = refuse to answer	ABCD Youth Mid-Year Phone Interview Substance Use	abcd_ymypisu01
	If child has used over the counter cough or cold medicine or DXM to get high 5 or more times in the past 6 months	mypi_cold_pst_6mo	1 = yes; 0 = no; 777 = refuse to answer		
	If child has used over the counter cough or cold medicine or DXM to get high in the past month	mypi_cold_pst_mo	1 = yes; 0 = no; 777 = refuse to answer		
	If child has used over the counter cough or cold medicine or DXM to get high in the past week	mypi_cold_lst_wk	1 = yes; 0 = no; 777 = refuse to answer		
	If child has sniffed liquids, sprays, gases, or other products to get high in the past 6 months	mypi_sniff_used	1 = yes; 0 = no; 777 = refuse to answer		
	If child sniffed liquids, sprays, or other products to get high 5 or more times in the past 6 months	mypi_sniff_pst_6mo	1 = yes; 0 = no; 777 = refuse to answer		

© 2024 Lee PH et al. *JAMA Network Open.*

Variable	Description	ABCD field	ABCD Value Ranges	ABCD Instrument	ABCD file
	If child sniffed liquids, sprays or other products to get high 5 or more times in the past month	mypi_sniff_pst_mo	1 = yes; 0 = no; 777 = refuse to answer		
	If child sniffed liquids, sprays or other products to get high 5 or more times in the past week	mypi_sniff_lst_wk	1 = yes; 0 = no; 777 = refuse to answer		
Other Substance Use	If child has ever tried fake marijuana or synthetics (such as K2 and spice)	tlfb_mj_synth_use	0 = No; 1 = Yes	ABCD Youth Substance Use Interview	abcd_ysu02
	If child has ever tried cathinones (such as bath salts, drone, M-cat, MDVP, or meph)	tlfb_bsalts_use	0 = No; 1 = Yes		
	If child has ever tried Methamphetamine, meth, or crystal meth	tlfb_meth_use	0 = No; 1 = Yes		
	If child has ever tried Ecstasy, molly, or MDMA	tlfb_mdma_use	0 = No; 1 = Yes		
	If child has ever tried Ketamine or special K	tlfb_ket_use	0 = No; 1 = Yes		
	If child has ever tried hallucinogen drugs (such as acid, LSD, PCP, angel dust, peyote, mescaline, DMT, AMT, Foxy)	tlfb_hall_use	0 = No; 1 = Yes		
	If child has ever tried hallucinogen drugs (such as magic mushrooms or shrooms)	tlfb_shrooms_use	0 = No; 1 = Yes		
	If child has ever tried hallucinogen drugs (such as salvia)	tlfb_salvia_use	0 = No; 1 = Yes		
	Which of these has child used? Check all that apply. (LSD or acid)	tlfb_hall_use_type___1	0 = No; 1 = Yes		
	Which of these has child used? Check all that apply. (PCP or angel dust)	tlfb_hall_use_type___2	0 = No; 1 = Yes		

© 2024 Lee PH et al. *JAMA Network Open.*

Variable	Description	ABCD field	ABCD Value Ranges	ABCD Instrument	ABCD file
	Which of these has child used? Check all that apply. (Peyote)	tlfb_hall_use_type___3	0 = No; 1 = Yes		
	Which of these has child used? Check all that apply. (Mescaline)	tlfb_hall_use_type___4	0 = No; 1 = Yes		
	Which of these has child used? Check all that apply. (DMT)	tlfb_hall_use_type___6	0 = No; 1 = Yes		
	Which of these has child used? Check all that apply. (AMT)	tlfb_hall_use_type___7	0 = No; 1 = Yes		
	Which of these has child used? Check all that apply. (Foxy)	tlfb_hall_use_type___8	0 = No; 1 = Yes		
	Which of these has child used? Check all that apply. (Other)	tlfb_hall_use_type___10	0 = No; 1 = Yes		
	If child has ever tried the depressant drug GHB, liquid G, or Georgia Home Boy	tlfb_ghb_use	0 = No; 1 = Yes		
	If child has ever tried steroids (such as arnolds, pumpers, or roids)	tlfb_steroids_use	0 = No; 1 = Yes		
	If child has used cathinones (such as bath salts, drone, M-cat, MDVP, or meph) in the past 6 months	mypi_cathinones_used	1=yes; 0=no; 777=refuse to answer [event-name] = 42_month_follow_up_arm_1" OR [event-name] = '54_month_follow_up_arm_1'"	ABCD Youth Mid-Year Phone Interview Substance Use	abcd_ymypisu01

© 2024 Lee PH et al. *JAMA Network Open.*

Variable	Description	ABCD field	ABCD Value Ranges	ABCD Instrument	ABCD file
	If child has used Methamphetamine, meth, or crystal meth in the past 6 months	mypi_meth_used	1=yes; 0=no; 777=refuse to answer [event-name] = "30_month_follow_up_arm_1" or [event-name] = "42_month_follow_up_arm_1" OR [event-name] = '54_month_follow_up_arm_1'		
	If child has used Ketamine or special K in the past 6 months	mypi_ketamine_used	1=yes; 0=no; 777=refuse to answer [event-name] = 42_month_follow_up_arm_1" OR [event-name] = '54_month_follow_up_arm_1'"		
	If child has used hallucinogen drugs (such as acid, LSD, PCP, angel dust, peyote, mescaline, DMT, AMT, Foxy) in the past 6 months	mypi_hallucinogen_used	1=yes; 0=no; 777=refuse to answer [event-name] = 42_month_follow_up_arm_1" OR [event-name] = '54_month_follow_up_arm_1'"		

© 2024 Lee PH et al. *JAMA Network Open.*

Variable	Description	ABCD field	ABCD Value Ranges	ABCD Instrument	ABCD file
	If child has used hallucinogen drugs (such as magic mushrooms or shrooms) in the past 6 months	mypi_mushroom_used	1=yes; 0=no; 777=refuse to answer [event-name] = 42_month_follow_up_arm_1" OR [event-name] = '54_month_follow_up_arm_1'"		
	If child has used the depressant drug GHB, liquid G, or Georgia Home Boy in the past 6 months	mypi_ghb_used	1=yes; 0=no; 777=refuse to answer [event-name] = 42_month_follow_up_arm_1" OR [event-name] = '54_month_follow_up_arm_1'"		
	If child has used steroids or roids in the past 6 months	mypi_steroids_used	1=yes; 0=no; 777=refuse to answer [event-name] = 42_month_follow_up_arm_1" OR [event-name] = '54_month_follow_up_arm_1'"		

© 2024 Lee PH et al. *JAMA Network Open.*

Variable	Description	ABCD field	ABCD Value Ranges	ABCD Instrument	ABCD file
	If child has used hallucinogen drugs (such as salvia) in the past 6 months	mypi_salvia_used	1=yes; 0=no; 777=refuse to answer [event-name] = 42_month_follow_up_arm_1" OR [event-name] = '54_month_follow_up_arm_1'"		

[a] To evaluate the specificity of the relationship between smoking tobacco products and risk of suicidal behavior outcomes, we compared the analysis results with those of alcohol sipping, alcohol drinking, cannabis and prescription use data generated based on the following table.

© 2024 Lee PH et al. *JAMA Network Open.*

eTable 8. Major Characteristics of ABCD Study Participants Based on Youth-Reported Ever Use of Tobacco Products in the 6-Month Follow-Up[a]

	**6 Month Assessment**		
	**Cases**	**Controls**	**p-value**
	n=123(1.37)	n=8865(98.63)	
**Age, Years, Mean (SD)**	10.54(0.62)	10.41(0.62)	2.43E-02
**Sex, n (%)**			
Male	74(60.16)	4613(52.04)	8.90E-02
Female	49(39.84)	4252(47.96)	
**Race, n (%)**			
Asian	1(0.81)	210(2.37)	6.38E-02
Black	18(14.63)	1219(13.75)	
Hispanic	20(16.26)	1747(19.71)	
Other / Mixed	22(17.89)	918(10.36)	
White	62(50.41)	4771(53.82)	
**Parental Education, n (%)**			
< HS Diploma / GED	7(5.69)	344(3.88)	3.00E-07
HS Diploma / GED	19(15.45)	756(8.53)	
Some College	52(42.28)	2260(25.49)	
Bachelor's Degree	25(20.33)	2296(25.90)	
Postgraduate Degree	20(16.26)	3209(36.20)	
**Household Income, n (%)**			
< $50,000	49(39.84)	2074(23.40)	1.37E-06
> $50,000 and < $100,000	39(31.71)	2319(26.16)	
> $100,000	35(28.46)	4472(50.45)	
**Marital Status of Parents, n (%)**			
Not Married	61(49.59)	2752(31.04)	1.65E-05
Married	62(50.41)	6113(68.96)	
**Parental History, n (%)**			
parental_alc_p_any	38(30.89)	1279(14.43)	5.72E-07
parental_dg_p_any	52(42.28)	2681(30.24)	5.39E-03
parental_dprs_p_any	36(29.27)	942(10.63)	1.13E-10
parental_trb_p_any	36(29.27)	1138(12.84)	1.64E-07
parental_prf_p_any	64(52.03)	3507(39.56)	6.64E-03
parental_scd_p_any	19(15.45)	475(5.36)	2.92E-06
**Prenatal Exposure, n (%)**			
early_tobacco	48(39.02)	1184(13.36)	6.04E-16
late_tobacco	27(21.95)	457(5.16)	1.30E-15

© 2024 Lee PH et al. *JAMA Network Open.*

[a] Information about the demographic, socioeconomic, and family history was obtained from the ABCD Parent Demographic Survey data. Cases represent children who acknowledged the use of tobacco products in the past or at present or had positive cotinine results from hair toxicological tests, while controls did not. P-values were from chi-square tests for categorical variables (e.g., sex) and unpaired t-test for quantitative measures (e.g., age). Significant p-values after multiple testing correction were marked usingsuperscripta.SD: standard deviation, HS: high school. Other in Race represents study participants who selected either multiple races, American Indian and Alaska Native, Native Hawaiian and Other Pacific, or Others in the survey.

© 2024 Lee PH et al. *JAMA Network Open.*

eTable 9. Major Characteristics of ABCD Study Participants Based on Youth-Reported Ever Use of Tobacco Products in the 18-Month Follow-Up[a]

	18 Month Assessment		
	**Cases**	**Controls**	**p-value**
	n=151(1.68)	n=8837(98.32)	
**Age, Years, Mean (SD)**	11.54(0.63)	11.39(0.62)	3.61E-03
**Sex, n (%)**			
Male	89(58.94)	4598(52.03)	1.09E-01
Female	62(41.06)	4239(47.97)	
**Race, n (%)**			
Asian	1(0.66)	210(2.38)	1.12E-01
Black	26(17.22)	1211(13.70)	
Hispanic	26(17.22)	1741(19.70)	
Other/Mixed	23(15.23)	917(10.38)	
White	75(49.67)	4758(53.84)	
**Parental Education, n (%)**			
< HS diploma/GED	8(5.30)	343(3.88)	1.68E-08
HS diploma / GED	24(15.89)	751(8.50)	
Some college	63(41.72)	2249(25.45)	
Bachelor's degree	29(19.21)	2292(25.94)	
Postgraduate degree	27(17.88)	3202(36.23)	
**Household Income, n (%)**			
< $50,000	59(39.07)	2064(23.36)	6.59E-07
> $50,000 and < $100,000	48(31.79)	2310(26.14)	
> $100,000	44(29.14)	4463(50.50)	
**Marital Status of Parents, n (%)**			
not Married	76(50.33)	2737(30.97)	9.52E-06
Married	75(49.67)	6100(69.03)	
**Parental History, n (%)**			
parental_alc_p_any	55(36.42)	2678(30.30)	9.00E-07
parental_dg_p_any	55(36.42)	2678(30.30)	1.26E-01
parental_dprs_p_any	43(28.48)	935(10.58)	6.39E-12
parental_trb_p_any	40(26.49)	1134(12.83)	1.46E-06
parental_prf_p_any	71(47.02)	3500(39.61)	7.08E-02
parental_scd_p_any	20(13.25)	474(5.36)	5.50E-05
**Prenatal Exposure, n (%)**			
early_tobacco	55(36.42)	1177(13.32)	7.25E-16
late_tobacco	30(19.87)	454(5.14)	7.87E-15

© 2024 Lee PH et al. *JAMA Network Open.*

[a] Information about the demographic, socioeconomic, and family history was obtained from the ABCD Parent Demographic Survey data. Cases represent children who acknowledged the use of tobacco products in the past or at present or had positive cotinine results from hair toxicological tests, while controls did not. P-values were from chi-square tests for categorical variables (e.g., sex) and unpaired t-test for quantitative measures (e.g., age). Significant p-values after multiple testing correction were marked usingsuperscripta.SD: standard deviation, HS: high school. Other in Race represents study participants who selected either multiple races, American Indian and Alaska Native, Native Hawaiian and Other Pacific, or Others in the survey.

© 2024 Lee PH et al. *JAMA Network Open.*

eTable 10. Association Between UTPs and Suicide Risk Outcomes Based on the Basic Model[a]

O	OA	P	PA	df	Case #	R^2 (%)	LRT P-value	OR	L95	H95	p-value	FDR	Sig
SA	Baseline	UTP	Baseline	8987	153	1.57	2.67E-06	6.97	3.61	13.44	7.13E-09	3.21E-08	****
SI	Baseline	UTP	Baseline	8834	1151	0.26	0.000417	2.48	1.55	3.96	1.50E-04	3.86E-04	***
NSSI	Baseline	UTP	Baseline	7683	428	0	0.891	1.07	0.39	2.98	8.90E-01	9.33E-01	
SA	Year 1	UTP	Baseline	8987	227	1.51	1.05E-07	6.58	3.71	11.67	1.11E-10	9.99E-10	****
SI	Year 1	UTP	Baseline	8760	1454	0.24	0.000473	2.37	1.5	3.74	2.13E-04	4.79E-04	***
NSSI	Year 1	UTP	Baseline	7306	508	0	0.933	0.96	0.34	2.67	9.33E-01	9.33E-01	
SA	Year 1	UTP	6 month	8987	227	1.62	3.77E-08	6.15	3.6	10.51	3.10E-11	5.58E-10	****
SI	Year 1	UTP	6 month	8760	1454	0.21	0.000845	2.13	1.4	3.24	4.24E-04	8.48E-04	***
NSSI	Year 1	UTP	6 month	7306	508	0	0.756	1.15	0.49	2.66	7.51E-01	9.01E-01	
SA	Year 2	UTP	Baseline	8987	321	0.83	9.31E-06	4.49	2.55	7.92	1.99E-07	5.97E-07	****
SI	Year 2	UTP	Baseline	8666	1853	0.13	0.00631	1.91	1.22	2.99	4.56E-03	8.21E-03	**
NSSI	Year 2	UTP	Baseline	6813	560	0	0.866	1.08	0.43	2.74	8.65E-01	9.33E-01	
SA	Year 2	UTP	6 month	8987	321	0.88	5.26E-06	4.21	2.48	7.15	1.05E-07	3.78E-07	****
SI	Year 2	UTP	6 month	8666	1853	0.1	0.0158	1.69	1.12	2.56	1.25E-02	1.88E-02	*
NSSI	Year 2	UTP	6 month	6813	560	0.01	0.665	1.19	0.54	2.63	6.58E-01	8.46E-01	
SA	Year 2	UTP	18 month	8987	321	1.21	9.46E-08	4.48	2.8	7.18	4.54E-10	2.72E-09	****
SI	Year 2	UTP	18 month	8666	1853	0.11	0.014	1.63	1.12	2.38	1.11E-02	1.82E-02	*
NSSI	Year 2	UTP	18 month	6813	560	0.04	0.303	1.44	0.74	2.81	2.81E-01	3.89E-01	

[a] Multivariate logistic regression was conducted to examine the association between UTPs and suicidal behavior outcomes, NSSI, SI, and SAs. Both exposure and outcome measures were binary variables. First, we examined a basic regression model, in which UTPs and each suicidal behavior outcome were tested as an independent predictor and a dependent variable, respectively, while controlling for demographic variables (age, sex, race). Statistically significant effects after multiple testing correction were marked on the plot using asterisks (false discovery rate (FDR)<0.001: ***, FDR <0.01: **, FDR<0.05: *) O: Outcome; OA: Outcome Assessment; P: Predictor; PA: Predictor Assessment; df: degree of freedom from multivariate logistic regression; R^2: *Nagelkerke's* Pseudo R^2**;** LRT: Likelihood Ratio Test**;** OR: Odds Ratio; L95, H95: 95% confidence lower, upper interval; FDR: false discovery rates; Sig: significance

© 2024 Lee PH et al. *JAMA Network Open.*

eTable 11. Association Between UTPs and Suicide Risk Outcomes Based on the All Covariate-Adjusted Model[a]

O	OA	P	PA	df	Case #	R^2 (%)	LRT P-Value	OR	L95	H95	p-value	FDR	Sig
SA	Baseline	UTP	Baseline	8987	153	1.06	0.000149	4.67	2.35	9.28	1.09E-05	4.90E-05	****
SI	Baseline	UTP	Baseline	8834	1151	0.16	0.0065	2.03	1.25	3.29	4.22E-03	9.06E-03	**
NSSI	Baseline	UTP	Baseline	7683	428	0	0.818	0.89	0.32	2.49	8.21E-01	9.27E-01	
SA	Year 1	UTP	Baseline	8987	227	0.96	3.29E-05	4.25	2.33	7.74	2.20E-06	1.98E-05	****
SI	Year 1	UTP	Baseline	8760	1454	0.15	0.00546	2	1.25	3.2	3.77E-03	9.06E-03	**
NSSI	Year 1	UTP	Baseline	7306	508	0	0.741	0.84	0.3	2.37	7.47E-01	9.27E-01	
SA	Year 1	UTP	6 month	8987	227	1.09	9.48E-06	4.24	2.42	7.43	4.15E-07	7.47E-06	****
SI	Year 1	UTP	6 month	8760	1454	0.14	0.00645	1.87	1.21	2.87	4.53E-03	9.06E-03	**
NSSI	Year 1	UTP	6 month	7306	508	0	0.917	1.05	0.45	2.45	9.17E-01	9.36E-01	
SA	Year 2	UTP	Baseline	8987	321	0.44	0.00159	2.85	1.58	5.13	4.86E-04	1.46E-03	**
SI	Year 2	UTP	Baseline	8666	1853	0.07	0.0424	1.63	1.03	2.59	3.73E-02	6.71E-02	
NSSI	Year 2	UTP	Baseline	6813	560	0	0.935	0.96	0.38	2.45	9.36E-01	9.36E-01	
SA	Year 2	UTP	6 month	8987	321	0.49	8.00E-04	2.83	1.63	4.9	2.08E-04	7.49E-04	***
SI	Year 2	UTP	6 month	8666	1853	0.06	0.0711	1.49	0.98	2.28	6.46E-02	9.69E-02	
NSSI	Year 2	UTP	6 month	6813	560	0	0.826	1.09	0.49	2.42	8.24E-01	9.27E-01	
SA	Year 2	UTP	18 month	8987	321	0.76	3.19E-05	3.17	1.95	5.17	3.56E-06	2.14E-05	****
SI	Year 2	UTP	18 month	8666	1853	0.06	0.0627	1.46	0.99	2.15	5.67E-02	9.28E-02	
NSSI	Year 2	UTP	18 month	6813	560	0.02	0.433	1.32	0.67	2.59	4.17E-01	5.77E-01	

[a] In the full covariate-adjusted model, we included sociodemographic, family history, and prenatal substance use variables which were identified as significant correlates of UTPs in preceding bivariate analyses, as covariates in addition to demographic data. Statistically significant effects after multiple testing correction were marked on the plot using asterisk (false discovery rate (FDR)<0.001: ***, FDR <0.01: **, FDR<0.05: *) O: Outcome; OA: Outcome Assessment; P: Predictor; PA: Predictor Assessment; df: degree of freedom from multivariate logistic regression; R^2: *Nagelkerke's* Pseudo R^2; LRT: Likelihood Ratio Test; OR: Odds Ratio; L95, H95: 95% confidence lower, upper interval; FDR: false discovery rates; Sig: significance

© 2024 Lee PH et al. *JAMA Network Open.*

eTable 12. Association Between UTPs and KSADS-5 Youth-Report-Based Suicide Risk Outcomes Based on the All Covariate-Adjusted Model[a]

O	OA	P	PA	df	Case #	R^2 (%)	LRT p-value	OR	L95	H95	p-value	FDR	Sig
SA	Baseline	UTP	Baseline	8987	125	1.33	8.35E-05	5.48	2.67	11.25	3.56E-06	1.60E-05	****
SI	Baseline	UTP	Baseline	8862	680	0.33	0.000467	2.8	1.66	4.74	1.22E-04	3.66E-04	***
NSSI	Baseline	UTP	Baseline	8182	319	0.04	0.331	1.64	0.64	4.15	3.00E-01	3.86E-01	
SA	Year 1	UTP	Baseline	8987	202	1.06	2.74E-05	4.57	2.46	8.47	1.41E-06	1.27E-05	****
SI	Year 1	UTP	Baseline	8785	1057	0.21	0.0018	2.31	1.41	3.77	8.59E-04	1.93E-03	**
NSSI	Year 1	UTP	Baseline	7728	418	0.01	0.589	1.3	0.51	3.32	5.76E-01	6.10E-01	
SA	Year 1	UTP	6 month	8987	202	1.23	6.37E-06	4.61	2.59	8.19	1.94E-07	3.49E-06	****
SI	Year 1	UTP	6 month	8785	1057	0.17	0.00507	2.01	1.27	3.19	2.91E-03	5.82E-03	**
NSSI	Year 1	UTP	6 month	7728	418	0.01	0.634	1.24	0.53	2.89	6.25E-01	6.25E-01	
SA	Year 2	UTP	Baseline	8987	275	0.54	0.000824	3.18	1.73	5.82	1.83E-04	4.71E-04	***
SI	Year 2	UTP	Baseline	8712	1315	0.15	0.00667	2.01	1.24	3.23	4.33E-03	7.79E-03	**
NSSI	Year 2	UTP	Baseline	7397	454	0.02	0.438	1.43	0.6	3.38	4.18E-01	5.02E-01	
SA	Year 2	UTP	6 month	8987	275	0.63	0.000324	3.19	1.82	5.61	5.57E-05	2.01E-04	***
SI	Year 2	UTP	6 month	8712	1315	0.1	0.0225	1.72	1.1	2.7	1.71E-02	2.80E-02	*
NSSI	Year 2	UTP	6 month	7397	454	0.02	0.524	1.31	0.59	2.89	5.10E-01	5.74E-01	
SA	Year 2	UTP	18 month	8987	275	0.86	2.60E-05	3.41	2.05	5.68	2.23E-06	1.34E-05	****
SI	Year 2	UTP	18 month	8712	1315	0.08	0.0525	1.54	1.01	2.34	4.42E-02	6.63E-02	
NSSI	Year 2	UTP	18 month	7397	454	0.1	0.109	1.75	0.92	3.34	8.72E-02	1.21E-01	

[a] In the full covariate-adjusted model, we included sociodemographic, family history, and prenatal substance use variables which were identified as significant correlates of UTPs in preceding bivariate analyses, as covariates in addition to demographic data. Statistically significant effects after multiple testing correction were marked on the plot using asterisk (false discovery rate (FDR)<0.001: ***, FDR <0.01: **, FDR<0.05: *) O: Outcome; OA: Outcome Assessment; P: Predictor; PA: Predictor Assessment; df: degree of freedom from multivariate logistic regression; R^2: *Nagelkerke's* Pseudo R^2**;** LRT: Likelihood Ratio Test**;** OR: Odds Ratio; L95, H95: 95% confidence lower, upper interval; FDR: false discovery rates; Sig: significance

© 2024 Lee PH et al. *JAMA Network Open.*

eTable 13. Association Between UTPs and KSADS-5 Parent-Report-Based Suicide Risk Outcomes Based on the All Covariate-Adjusted Model[a]

O	OA	P	PA	df	Case #	R^2 (%)	LRT p-value	OR	L95	H95	p-value	FDR	Sig
SA	Baseline	UTP	Baseline	8987	40	1.22	0.0183	5.04	1.59	15.95	5.94E-03	3.56E-02	*
SI	Baseline	UTP	Baseline	8947	678	0.03	0.265	1.43	0.78	2.61	2.48E-01	5.95E-01	
NSSI	Baseline	UTP	Baseline	8269	211	0	0.963	0.97	0.3	3.17	9.63E-01	9.63E-01	
SA	Year 2	UTP	Baseline	8987	85	0.23	0.168	2.32	0.78	6.88	1.29E-01	3.87E-01	
SI	Year 2	UTP	Baseline	8902	1048	0	0.869	1.05	0.6	1.85	8.68E-01	9.63E-01	
NSSI	Year 2	UTP	Baseline	7854	324	0	0.773	1.15	0.45	2.92	7.69E-01	9.63E-01	
SA	Year 2	UTP	6 month	8987	85	0.62	0.0223	3.32	1.34	8.23	9.55E-03	3.82E-02	*
SI	Year 2	UTP	6 month	8902	1048	0	0.939	1.02	0.6	1.73	9.39E-01	9.63E-01	
NSSI	Year 2	UTP	6 month	7854	324	0.03	0.401	1.43	0.65	3.15	3.80E-01	6.79E-01	

[a] In the full covariate-adjusted model, we included sociodemographic, family history, and prenatal substance use variables which were identified as significant correlates of UTPs in preceding bivariate analyses, as covariates in addition to demographic data. Statistically significant effects after multiple testing correction were marked on the plot using asterisk (false discovery rate (FDR)<0.001: ***, FDR <0.01: **, FDR<0.05: *) O: Outcome; OA: Outcome Assessment; P: Predictor; PA: Predictor Assessment; df: degree of freedom from multivariate logistic regression; R^2: *Nagelkerke's* Pseudo R^2**;** LRT: Likelihood Ratio Test**;** OR: Odds Ratio; L95, H95: 95% confidence lower, upper interval; FDR: false discovery rates; Sig: significance

© 2024 Lee PH et al. *JAMA Network Open.*

eTable 14. Association between UTPs and Suicide Risk Outcome Measures Concordant Between KSAS-5 Youth- and Parent-Reports Assessed in the All Covariate-Adjusted Model[a]

O	OA	P	PA	df	Case #	R^2 (%)	LRT p-value	OR	L95	H95	p-value	FDR	Sig
SA	Baseline	UTP	Baseline	8987	12	7.33	0.001	24.51	4.99	120.31	8.09E-05	9.71E-04	***
SI	Baseline	UTP	Baseline	8975	204	0.35	0.0159	2.96	1.35	6.51	6.95E-03	1.39E-02	*
NSSI	Baseline	UTP	Baseline	8771	59	1.02	0.00982	5.62	1.91	16.57	1.76E-03	1.06E-02	*
SA	Year 2	UTP	Baseline	8987	28	1.8	0.0146	7.56	1.95	29.37	3.48E-03	1.39E-02	*
SI	Year 2	UTP	Baseline	8959	360	0.14	0.0589	2.07	1.03	4.14	4.12E-02	4.12E-02	*
NSSI	Year 2	UTP	Baseline	8599	107	0.47	0.0275	3.49	1.33	9.13	1.10E-02	1.85E-02	*
SA	Year 2	UTP	6 month	8987	28	1.57	0.0226	6.28	1.66	23.7	6.72E-03	1.39E-02	*
SI	Year 2	UTP	6 month	8959	360	0.18	0.0324	2.12	1.12	4	2.03E-02	2.44E-02	*
NSSI	Year 2	UTP	6 month	8599	107	0.38	0.0487	2.97	1.15	7.68	2.47E-02	2.69E-02	*
SA	Year 2	UTP	18 month	8987	28	1.29	0.0384	5.04	1.37	18.56	1.50E-02	2.00E-02	*
SI	Year 2	UTP	18 month	8959	360	0.26	0.0103	2.26	1.28	4	5.17E-03	1.39E-02	*
NSSI	Year 2	UTP	18 month	8599	107	0.47	0.0281	3.04	1.27	7.26	1.23E-02	1.85E-02	*

[a] In the full covariate-adjusted model, we included sociodemographic, family history, and prenatal substance use variables which were identified as significant correlates of UTPs in preceding bivariate analyses, as covariates in addition to demographic data. Statistically significant effects after multiple testing correction were marked on the plot using asterisk (false discovery rate (FDR)<0.001: ***, FDR <0.01: **, FDR<0.05: *) O: Outcome; OA: Outcome Assessment; P: Predictor; PA: Predictor Assessment; df: degree of freedom from multivariate logistic regression; R^2: *Nagelkerke's* Pseudo R^2**;** LRT: Likelihood Ratio Test**;** OR: Odds Ratio; L95, H95: 95% confidence lower, upper interval; FDR: false discovery rates, Sig: significance

© 2024 Lee PH et al. *JAMA Network Open.*

eTable 15. Associations of the Use of Tobacco Products (UTPs) and New SA Cases, While Adjusting for Various Confounding Factors

Regression Model	UTP Assessment	New SA Assessment	df	Case #	R^2 (%)	LRT p-value	Estimate	SE	OR	L95	H95	p.value
Basic Model	Baseline	Year 1	8834	74	0.81	1.06E-02	1.665	0.529	5.29	1.87	14.92	1.66E-03
		Year 2	8834	168	0.13	1.68E-01	0.801	0.52	2.23	0.8	6.18	1.23E-01
	6 Month	Year 1	8834	74	1.48	5.46E-04	1.904	0.44	6.71	2.83	15.9	1.53E-05
		Year 2	8834	168	0.29	3.58E-02	1.032	0.429	2.81	1.21	6.51	1.62E-02
	18 Month	Year 2	8834	74	1.65	2.55E-04	1.859	0.41	6.42	2.88	14.32	5.64E-06
All Covariate-Adjusted Model	Baseline	Year 1	8834	74	0.49	5.35E-02	1.216	0.547	3.37	1.15	9.85	2.62E-02
		Year 2	8834	168	0.03	5.37E-01	0.342	0.53	1.41	0.5	3.98	5.19E-01
	6 Month	Year 1	8834	74	1.08	4.01E-03	1.553	0.456	4.72	1.93	11.55	6.65E-04
		Year 2	8834	168	0.12	1.78E-01	0.639	0.439	1.9	0.8	4.48	1.45E-01
	18 Month	Year 2	8834	168	0.24	6.06E-02	0.791	0.383	2.21	1.04	4.67	3.91E-02

[a] New SA cases were defined as those who did not report lifetime SAs in the baseline but did in the outcome assessment time in year 1 and Year 2. In a basic model, we included age, sex, and parent-reported race/ethnicity as covariates. In all covariate-adjusted model, we additionally included sociodemographic, family history, and prenatal substance use variables which were identified as significant correlates of UTPs. O: Outcome; OA: Outcome Assessment; P: Predictor; PA: Predictor Assessment; df: degree of freedom from multivariate logistic regression; R^2: *Nagelkerke's* Pseudo R^2**;** LRT: Likelihood Ratio Test**;** Estimate: beta estimate from regression; SE: standard error; OR: Odds Ratio; L95, H95: 95% confidence lower, upper intervals

© 2024 Lee PH et al. *JAMA Network Open.*

eTable 16. Multivariate Logistic Regression Results for Predicting Suicide Attempts Using UTPs (Both Lifetime Measures Assessed in the Baseline)[a]

Category	Predictor	Multivariate Logistic Regression				Likelihood Ratio Test	
		OR	95% CI	P-value	R^2 (%)	P-value	FDR
Demographics	Age	1.02	0.83-1.26	8.22E-01	0.01	8.22E-01	1.00E+00
	Female	0.83	0.54-1.26	3.70E-01	0.1	3.68E-01	7.79E-01
	Race (Asian)	2.66	0.78-9.12	1.19E-01	0	1.00E+00	1.00E+00
	Race (Black)	1.82	0.95-3.48	6.95E-02	0	1.00E+00	1.00E+00
	Race (Hispanic)	1.17	0.65-2.10	6.05E-01	0	1.00E+00	1.00E+00
	Race (Other/Mixed)	1.32	0.69-2.49	4.01E-01	0	1.00E+00	1.00E+00
Socioeconomic Factors	Income	1.04	0.79-1.38	7.72E-01	0.01	7.71E-01	1.00E+00
	Married	0.66	0.41-1.06	8.70E-02	0.34	8.83E-02	4.54E-01
	Parental Education (HS Diploma / GED)	1.56	0.46-5.29	4.78E-01	0	1.00E+00	1.00E+00
	Parental Education (Some College)	1.65	0.54-5.06	3.81E-01	0	1.00E+00	1.00E+00
	Parental Education (Bachelor's Degree)	1.59	0.48-5.29	4.53E-01	0	1.00E+00	1.00E+00
	Parental Education (Postgraduate)	1.18	0.34-4.17	7.93E-01	0	1.00E+00	1.00E+00
Prenatal History	Early Tobacco	0.77	0.34-1.77	5.40E-01	0.04	5.39E-01	1.00E+00
	Early Exposure No	1.2	0.91-1.58	2.04E-01	0.18	2.13E-01	6.87E-01
	Late Tobacco	1.03	0.25-4.37	9.63E-01	0	9.63E-01	1.00E+00
	Late Exposure No	0.89	0.63-1.27	5.37E-01	0.05	5.16E-01	1.00E+00
Parental History	Alcohol Problems	0.55	0.30-1.01	5.48E-02	0.47	4.63E-02	2.78E-01
	Depression	1	0.53-1.89	9.96E-01	0	9.96E-01	1.00E+00
	Troubles	1.88	1.05-3.36	3.36E-02	0.51	3.73E-02	2.69E-01
	Suicide	2.28	1.25-4.18	7.27E-03	0.76	1.12E-02	1.21E-01
Child Psychopathology	Rule-Breaking Behaviors	1.01	0.80-1.26	9.61E-01	0	9.61E-01	1.00E+00
	Social Problems	1.14	0.92-1.40	2.25E-01	0.17	2.29E-01	6.87E-01
	Aggressive Behaviors	1.07	0.83-1.40	5.91E-01	0.03	5.93E-01	1.00E+00
	Attention Problems	1.13	0.93-1.37	2.22E-01	0.17	2.28E-01	6.87E-01
	Anxious/Depressed	1.11	0.89-1.39	3.49E-01	0.1	3.54E-01	7.79E-01

© 2024 Lee PH et al. *JAMA Network Open.*

Category	Predictor	Multivariate Logistic Regression				Likelihood Ratio Test	
		OR	95% CI	P-value	R^2 (%)	P-value	FDR
	Withdrawn/Depressed	1.12	0.91-1.37	2.82E-01	0.13	2.89E-01	7.79E-01
DSM-V	Conduct Disorder	0.55	0.15-1.96	3.52E-01	0.11	3.45E-01	7.79E-01
	Oppositional Defiant Disorder	0.47	0.15-1.45	1.90E-01	0.22	1.78E-01	6.87E-01
Temperaments	Negative Urgency	1.32	1.06-1.64	1.28E-02	0.72	1.34E-02	1.21E-01
	Lack of Planning	1.35	1.09-1.67	6.57E-03	0.84	7.59E-03	1.21E-01
	Positive Urgency	1.11	0.90-1.37	3.25E-01	0.11	3.25E-01	7.79E-01
	Lack Perseverance	1.02	0.83-1.27	8.28E-01	0.01	8.28E-01	1.00E+00
	Sensation Seeking	0.87	0.70-1.08	2.01E-01	0.19	1.99E-01	6.87E-01
	Aggression	1	0.81-1.23	9.86E-01	0	9.86E-01	1.00E+00
	Depressive Mood	0.99	0.80-1.23	9.40E-01	0	9.40E-01	1.00E+00
Substance Use	**Use of Tobacco Products**	**4.34**	**2.06-9.13**	**1.13E-04**	**1.39**	**5.72E-04**	**2.06E-02**

[a] All listed predictors were examined simultaneously in the regression model. Likelihood ratio tests were conducted by comparing the full regression model with the same model without the corresponding predictor. Predictors with likelihood ratio test FDR<5% are marked in bold. OR: Odds Ratio, CI: confidence interval, LR Test: likelihood ratio test. R^2: *Nagelkerke's* Pseudo R^2

© 2024 Lee PH et al. *JAMA Network Open.*

eTable 17. Multivariate Logistic Regression Results for Predicting Suicide Attempts Using UTPs (UTPs Assessed in 6-Month Follow-Up, SA Assessed in Year 1 Follow-Up)[a]

Category	Predictor	Multivariate Logistic Regression				Likelihood Ratio Test	
		OR	95% CI	P-value	R^2 (%)	P-value	FDR
Demographics	Age	1.16	0.97-1.38	9.43E-02	0.26	9.36E-02	4.29E-01
	Female	0.78	0.54-1.11	1.63E-01	0.18	1.60E-01	5.76E-01
	Race (Asian)	1.62	0.48-5.46	4.35E-01	0	1.00E+00	1.00E+00
	Race (Black)	1.55	0.89-2.69	1.18E-01	0	1.00E+00	1.00E+00
	Race (Hispanic)	1.03	0.63-1.69	9.03E-01	0	1.00E+00	1.00E+00
	Race (Other/Mixed)	1.13	0.65-1.96	6.55E-01	0	1.00E+00	1.00E+00
Socioeconomic Factors	Income	1	0.79-1.26	9.80E-01	0	9.80E-01	1.00E+00
	**Married**	0.59	0.39-0.88	9.50E-03	0.61	9.81E-03	1.15E-01
	Parental Education (HS Diploma / GED)	1.28	0.48-3.39	6.24E-01	0	1.00E+00	1.00E+00
	Parental Education (Some College)	1.49	0.62-3.60	3.74E-01	0	1.00E+00	1.00E+00
	Parental Education (Bachelor's Degree)	1.21	0.46-3.16	6.99E-01	0	1.00E+00	1.00E+00
	Parental Education (Postgraduate)	1.02	0.37-2.76	9.73E-01	0	1.00E+00	1.00E+00
Prenatal History	Early Tobacco	0.71	0.35-1.43	3.41E-01	0.08	3.38E-01	8.28E-01
	Early Exposure No	1.22	0.97-1.54	8.91E-02	0.26	9.53E-02	4.29E-01
	Late Tobacco	0.82	0.27-2.47	7.23E-01	0.01	7.23E-01	1.00E+00
	Late Exposure No	0.99	0.78-1.26	9.50E-01	0	9.50E-01	1.00E+00
Parental History	Alcohol Problems	0.75	0.46-1.22	2.44E-01	0.13	2.36E-01	7.08E-01
	Depression	1.25	0.73-2.12	4.14E-01	0.06	4.17E-01	8.83E-01
	Troubles	1.11	0.67-1.85	6.90E-01	0.01	6.91E-01	1.00E+00
	Suicide	2.03	1.19-3.45	9.20E-03	0.57	1.28E-02	1.15E-01
Child Psychopathology	Rule-Breaking Behaviors	1.07	0.88-1.29	4.92E-01	0.04	4.95E-01	9.31E-01
	Social Problems	1.12	0.94-1.34	2.05E-01	0.15	2.08E-01	6.81E-01
	Aggressive Behaviors	1.11	0.89-1.38	3.64E-01	0.07	3.68E-01	8.28E-01
	Attention Problems	1.02	0.86-1.21	7.92E-01	0.01	7.93E-01	1.00E+00

© 2024 Lee PH et al. *JAMA Network Open.*

Category	Predictor	Multivariate Logistic Regression				Likelihood Ratio Test	
		OR	95% CI	P-value	R^2 (%)	P-value	FDR
	Anxious/Depressed	1.07	0.88-1.29	4.90E-01	0.04	4.93E-01	9.31E-01
	Withdrawn/Depressed	1.19	1.00-1.40	4.63E-02	0.35	5.12E-02	3.69E-01
DSM-V	Conduct Disorder	0.48	0.17-1.35	1.63E-01	0.18	1.56E-01	5.76E-01
	Oppositional Defiant Disorder	0.77	0.32-1.83	5.48E-01	0.03	5.45E-01	9.34E-01
Temperaments	**Negative Urgency**	**1.54**	**1.28-1.85**	**5.35E-06**	**1.86**	**5.98E-06**	**1.96E-04**
	Lack of Planning	1.18	0.98-1.42	7.83E-02	0.28	8.11E-02	4.29E-01
	Positive Urgency	1.06	0.89-1.27	5.16E-01	0.04	5.17E-01	9.31E-01
	Lack Perseverance	1.11	0.92-1.33	2.79E-01	0.11	2.81E-01	7.78E-01
	Sensation Seeking	0.92	0.76-1.10	3.45E-01	0.08	3.45E-01	8.28E-01
	Aggression	1.01	0.85-1.20	9.18E-01	0	9.18E-01	1.00E+00
	Depressive Mood	1.01	0.84-1.21	9.03E-01	0	9.03E-01	1.00E+00
Substance Use	**Use of Tobacco Products**	**4.61**	**2.49-8.52**	**1.14E-06**	**1.75**	**1.09E-05**	**1.96E-04**

[a] All listed predictors were examined simultaneously in the regression model. Likelihood ratio tests were conducted by comparing the full regression model with the same model without the corresponding predictor. Predictors with likelihood ratio test FDR<5% are marked in bold. OR: Odds Ratio, CI: confidence interval, LR Test: likelihood ratio test. R^2: *Nagelkerke's* Pseudo R^2

© 2024 Lee PH et al. *JAMA Network Open.*

eTable 18. Multivariate Logistic Regression Results for Predicting Suicidal Ideation Using UTPs (UTPs and SI Both Assessed in Baseline)[a]

Category	Predictor	Multivariate Logistic Regression				Likelihood Ratio Test	
		OR	95% CI	P-value	R^2 (%)	P-value	FDR
Demographics	Age	0.98	0.91-1.06	6.72E-01	0.01	6.72E-01	1.00E+00
	**Female**	**0.69**	**0.59-0.82**	**1.12E-05**	**0.64**	**9.75E-06**	**1.17E-04**
	Race (Asian)	1.88	1.18-2.99	7.49E-03	0	1.00E+00	1.00E+00
	Race (Black)	1.03	0.76-1.39	8.60E-01	0	1.00E+00	1.00E+00
	Race (Hispanic)	0.95	0.76-1.20	6.95E-01	0	1.00E+00	1.00E+00
	Race (Other/Mixed)	1.21	0.94-1.55	1.47E-01	0	1.00E+00	1.00E+00
Socioeconomic Factors	Income	1.07	0.95-1.20	2.46E-01	0.04	2.45E-01	5.51E-01
	Married	0.95	0.78-1.17	6.40E-01	0.01	6.40E-01	1.00E+00
	Parental Education (HS Diploma / GED)	1.55	0.87-2.78	1.39E-01	0	1.00E+00	1.00E+00
	Parental Education (Some College)	1.71	1.00-2.91	4.94E-02	0	1.00E+00	1.00E+00
	Parental Education (Bachelor's Degree)	1.6	0.92-2.80	9.69E-02	0	1.00E+00	1.00E+00
	Parental Education (Postgraduate)	1.73	0.98-3.05	5.71E-02	0	1.00E+00	1.00E+00
Prenatal History	Early Tobacco	1.04	0.73-1.49	8.34E-01	0	8.34E-01	1.00E+00
	Early Exposure No	1.05	0.94-1.18	3.83E-01	0.02	3.85E-01	8.15E-01
	Late Tobacco	0.54	0.30-0.99	4.52E-02	0.13	4.44E-02	1.78E-01
	Late Exposure No	1.05	0.93-1.19	4.52E-01	0.02	4.55E-01	8.19E-01
Parental History	Alcohol Problems	1.18	0.93-1.49	1.67E-01	0.06	1.70E-01	4.08E-01
	Depression	0.89	0.66-1.19	4.22E-01	0.02	4.20E-01	8.19E-01
	**Troubles**	**1.45**	**1.10-1.90**	**7.70E-03**	**0.23**	**8.36E-03**	**3.76E-02**
	Suicide	1.31	0.95-1.80	1.00E-01	0.09	1.06E-01	3.18E-01
Child Psychopathology	**Rule-Breaking Behaviors**	**1.24**	**1.12-1.37**	**2.87E-05**	**0.55**	**3.82E-05**	**3.44E-04**
	Social Problems	1.1	1.00-1.21	5.87E-02	0.12	5.98E-02	1.96E-01
	Aggressive Behaviors	1.04	0.93-1.17	5.01E-01	0.01	5.01E-01	8.59E-01
	Attention Problems	0.93	0.84-1.02	1.38E-01	0.07	1.35E-01	3.73E-01
	**Anxious/Depressed**	**1.28**	**1.17-1.41**	**7.03E-08**	**0.92**	**1.04E-07**	**3.74E-06**
	Withdrawn/Depressed	1.01	0.93-1.11	7.79E-01	0	7.80E-01	1.00E+00

© 2024 Lee PH et al. *JAMA Network Open.*

Category	Predictor	Multivariate Logistic Regression				Likelihood Ratio Test	
		OR	95% CI	P-value	R^2 (%)	P-value	FDR
DSM-V	Conduct Disorder	0.9	0.49-1.63	7.18E-01	0	7.18E-01	1.00E+00
	Oppositional Defiant Disorder	0.81	0.48-1.37	4.35E-01	0.02	4.34E-01	8.19E-01
Temperaments	**Negative Urgency**	**1.14**	**1.05-1.25**	**3.40E-03**	**0.28**	**3.51E-03**	**1.81E-02**
	Lack of Planning	1.09	1.00-1.19	5.58E-02	0.12	5.68E-02	1.96E-01
	Positive Urgency	1	0.91-1.09	9.17E-01	0	9.17E-01	1.00E+00
	**Lack Perseverance**	**1.22**	**1.12-1.33**	**7.82E-06**	**0.65**	**8.46E-06**	**1.17E-04**
	**Sensation Seeking**	**1.14**	**1.04-1.24**	**2.99E-03**	**0.29**	**2.97E-03**	**1.78E-02**
	Aggression	1.02	0.94-1.11	5.79E-01	0.01	5.78E-01	9.46E-01
	**Depressive Mood**	**0.84**	**0.77-0.92**	**6.70E-05**	**0.52**	**6.48E-05**	**4.67E-04**
Substance Use	Use of Tobacco Products	1.5	0.88-2.54	1.36E-01	0.07	1.45E-01	3.73E-01

[a] All listed predictors were examined simultaneously in the regression model. Likelihood ratio tests were conducted by comparing the full regression model with the same model without the corresponding predictor. Predictors with likelihood ratio test FDR<5% are marked in bold. OR: Odds Ratio, CI: confidence interval, LR Test: likelihood ratio test. R^2: *Nagelkerke's* Pseudo R^2

© 2024 Lee PH et al. *JAMA Network Open.*

eTable 19. Multivariate Logistic Regression Results for Predicting Suicidal Ideation Using UTPs (UTPs Assessed in 6-Month Follow-Up, SI Assessed in Year 1 Follow-Up)[a]

Category	Predictor	Multivariate Logistic Regression				Likelihood Ratio Test	
		OR	95% CI	P-value	R^2 (%)	P-value	FDR
Demographics	Age	0.98	0.91-1.05	5.32E-01	0.01	5.32E-01	9.12E-01
	Female	0.83	0.72-0.96	1.13E-02	0.19	1.11E-02	5.49E-02
	Race (Asian)	1.53	0.98-2.38	6.00E-02	0	1.00E+00	1.00E+00
	Race (Black)	1.23	0.95-1.61	1.19E-01	0	1.00E+00	1.00E+00
	Race (Hispanic)	0.98	0.80-1.21	8.80E-01	0	1.00E+00	1.00E+00
	Race (Other/Mixed)	1.16	0.92-1.47	2.04E-01	0	1.00E+00	1.00E+00
Socioeconomic Factors	Income	1.04	0.94-1.15	4.76E-01	0.02	4.76E-01	8.57E-01
	Married	1.03	0.85-1.24	7.83E-01	0	7.83E-01	1.00E+00
	Parental Education (HS Diploma / GED)	1.16	0.71-1.88	5.60E-01	0	1.00E+00	1.00E+00
	Parental Education (Some College)	1.24	0.80-1.92	3.43E-01	0	1.00E+00	1.00E+00
	Parental Education (Bachelor's Degree)	1.23	0.77-1.95	3.80E-01	0	1.00E+00	1.00E+00
	Parental Education (Postgraduate)	1.31	0.82-2.10	2.58E-01	0	1.00E+00	1.00E+00
Prenatal History	Early Tobacco	1	0.72-1.39	9.93E-01	0	9.93E-01	1.00E+00
	Early Exposure No	1.04	0.94-1.16	4.61E-01	0.02	4.62E-01	8.57E-01
	Late Tobacco	0.69	0.40-1.19	1.84E-01	0.05	1.84E-01	5.10E-01
	Late Exposure No	1.03	0.92-1.16	6.26E-01	0.01	6.27E-01	9.81E-01
Parental History	Alcohol Problems	1.18	0.95-1.46	1.31E-01	0.07	1.33E-01	4.35E-01
	Depression	0.99	0.75-1.29	9.21E-01	0	9.21E-01	1.00E+00
	Troubles	1.24	0.96-1.59	9.57E-02	0.08	9.77E-02	3.52E-01
	Suicide	1.47	1.09-1.98	1.07E-02	0.19	1.22E-02	5.49E-02
Child Psychopathology	**Rule-Breaking Behaviors**	**1.19**	**1.08-1.31**	**3.00E-04**	**0.38**	**3.51E-04**	**3.16E-03**
	Social Problems	1.05	0.96-1.15	2.48E-01	0.04	2.49E-01	5.60E-01
	Aggressive Behaviors	1.02	0.92-1.14	7.05E-01	0	7.05E-01	1.00E+00
	Attention Problems	0.96	0.87-1.04	3.14E-01	0.03	3.13E-01	6.63E-01
	**Anxious/Depresse d**	**1.29**	**1.19-1.41**	**1.72E-09**	**1.04**	**2.50E-09**	**9.00E-08**

© 2024 Lee PH et al. *JAMA Network Open.*

Category	Predictor	Multivariate Logistic Regression				Likelihood Ratio Test	
		OR	95% CI	P-value	R^2 (%)	P-value	FDR
	Withdrawn/Depressed	1.06	0.98-1.15	1.73E-01	0.05	1.75E-01	5.10E-01
DSM-V	Conduct Disorder	1.13	0.63-2.05	6.77E-01	0.01	6.77E-01	1.00E+00
	Oppositional Defiant Disorder	0.73	0.44-1.22	2.28E-01	0.04	2.26E-01	5.42E-01
Temperaments	**Negative Urgency**	**1.22**	**1.13-1.33**	**1.23E-06**	**0.69**	**1.32E-06**	**2.38E-05**
	**Lack of Planning**	**1.12**	**1.03-1.21**	**7.91E-03**	**0.21**	**8.15E-03**	**4.89E-02**
	Positive Urgency	1.02	0.94-1.11	6.06E-01	0.01	6.07E-01	9.81E-01
	**Lack Perseverance**	**1.19**	**1.10-1.29**	**1.74E-05**	**0.54**	**1.84E-05**	**2.21E-04**
	Sensation Seeking	1.1	1.01-1.18	1.94E-02	0.16	1.93E-02	7.72E-02
	Aggression	1.05	0.97-1.13	2.10E-01	0.05	2.10E-01	5.40E-01
	**Depressive Mood**	**0.88**	**0.82-0.95**	**1.45E-03**	**0.3**	**1.43E-03**	**1.03E-02**
Substance Use	Use of Tobacco Products	1.25	0.73-2.14	4.13E-01	0.02	4.20E-01	8.40E-01

[a] All listed predictors were examined simultaneously in the regression model. Likelihood ratio tests were conducted by comparing the full regression model with the same model without the corresponding predictor. Predictors with likelihood ratio test FDR<5% are marked in bold. OR: Odds Ratio, CI: confidence interval, LR Test: likelihood ratio test. R^2: *Nagelkerke's* Pseudo R^2

© 2024 Lee PH et al. *JAMA Network Open.*

eTable 20. Multivariate Logistic Regression Results for Predicting Suicidal Ideation Using UTPs (UTPs Assessed in 18-Month Follow-Up, SI Assessed in Year 2 Follow-Up)[a]

Category	Predictor	Multivariate Logistic Regression				Likelihood Ratio Test	
		OR	95% CI	P-value	R^2 (%)	P-value	FDR
Demographics	Age	1	0.94-1.07	9.94E-01	0	9.94E-01	1.00E+00
	Female	0.9	0.78-1.03	1.18E-01	0.07	1.18E-01	3.27E-01
	Race (Asian)	1.24	0.80-1.90	3.33E-01	0	1.00E+00	1.00E+00
	Race (Black)	1.15	0.89-1.47	2.78E-01	0	1.00E+00	1.00E+00
	Race (Hispanic)	0.94	0.77-1.14	5.06E-01	0	1.00E+00	1.00E+00
	Race (Other/Mixed)	1.12	0.90-1.39	3.21E-01	0	1.00E+00	1.00E+00
Socioeconomic Factors	Income	1.02	0.92-1.12	7.58E-01	0	7.58E-01	1.00E+00
	Married	1.07	0.90-1.27	4.65E-01	0.01	4.64E-01	8.35E-01
	Parental Education (HS Diploma / GED)	1.1	0.70-1.72	6.79E-01	0	1.00E+00	1.00E+00
	Parental Education (Some College)	1.13	0.75-1.68	5.60E-01	0	1.00E+00	1.00E+00
	Parental Education (Bachelor's Degree)	1.14	0.75-1.75	5.31E-01	0	1.00E+00	1.00E+00
	Parental Education (Postgraduate)	1.19	0.77-1.83	4.31E-01	0	1.00E+00	1.00E+00
Prenatal History	Early Tobacco	0.79	0.58-1.08	1.39E-01	0.06	1.37E-01	3.52E-01
	Early Exposure No	1.11	1.00-1.22	4.31E-02	0.11	4.39E-02	1.57E-01
	Late Tobacco	0.75	0.44-1.28	2.93E-01	0.03	2.93E-01	6.50E-01
	Late Exposure No	1.01	0.90-1.12	9.18E-01	0	9.18E-01	1.00E+00
Parental History	Alcohol Problems	1.09	0.89-1.34	3.86E-01	0.02	3.88E-01	7.60E-01
	Depression	1.12	0.86-1.44	3.99E-01	0.02	4.01E-01	7.60E-01
	Troubles	1.14	0.90-1.45	2.81E-01	0.03	2.82E-01	6.50E-01
	**Suicide**	**1.52**	**1.14-2.03**	**4.55E-03**	**0.22**	**5.18E-03**	**2.33E-02**
Child Psychopathology	**Rule-Breaking Behaviors**	**1.17**	**1.07-1.28**	**4.72E-04**	**0.33**	**5.23E-04**	**4.17E-03**
	Social Problems	1.09	1.00-1.19	4.75E-02	0.11	4.80E-02	1.57E-01
	Aggressive Behaviors	0.98	0.89-1.09	7.29E-01	0	7.29E-01	1.00E+00

© 2024 Lee PH et al. *JAMA Network Open.*

Category	Predictor	Multivariate Logistic Regression				Likelihood Ratio Test	
		OR	95% CI	P-value	R^2 (%)	P-value	FDR
	Attention Problems	0.99	0.91-1.07	7.25E-01	0	7.25E-01	1.00E+00
	**Anxious/Depressed**	**1.38**	**1.27-1.49**	**2.34E-15**	**1.72**	**2.50E-15**	**9.00E-14**
	**Withdrawn/Depressed**	**1.11**	**1.02-1.19**	**1.14E-02**	**0.18**	**1.19E-02**	**4.76E-02**
DSM-V	Conduct Disorder	1.02	0.55-1.90	9.56E-01	0	9.56E-01	1.00E+00
	Oppositional Defiant Disorder	0.76	0.45-1.28	3.07E-01	0.03	3.07E-01	6.50E-01
Temperaments	**Negative Urgency**	**1.29**	**1.19-1.39**	**1.18E-10**	**1.14**	**1.20E-10**	**2.16E-09**
	**Lack of Planning**	**1.14**	**1.06-1.23**	**7.54E-04**	**0.31**	**7.78E-04**	**4.17E-03**
	Positive Urgency	1.02	0.95-1.11	5.69E-01	0.01	5.69E-01	9.75E-01
	Lack Perseverance	1.22	1.13-1.31	2.83E-07	0.73	2.88E-07	3.46E-06
	Sensation Seeking	1.13	1.05-1.22	6.74E-04	0.32	6.63E-04	4.17E-03
	Aggression	1.06	0.99-1.14	8.87E-02	0.08	8.83E-02	2.65E-01
	**Depressive Mood**	**0.89**	**0.82-0.95**	**8.21E-04**	**0.31**	**8.10E-04**	**4.17E-03**
Substance Use	Use of Tobacco Products	1.03	0.61-1.76	9.04E-01	0	9.04E-01	1.00E+00

[a] All listed predictors were examined simultaneously in the regression model. Likelihood ratio tests were conducted by comparing the full regression model with the same model without the corresponding predictor. Predictors with likelihood ratio test FDR<5% are marked in bold. OR: Odds Ratio, CI: confidence interval, LR Test: likelihood ratio test. R^2: *Nagelkerke's* Pseudo R^2

© 2024 Lee PH et al. *JAMA Network Open.*

eTable 21. Interaction Analysis Results of UTP at Baseline with Children's Cognition, Temperament/Personality, and Psychopathology Measures on SA

OR: Odds Ratio; L95: Lower 95% Confidence Interval; H95: Upper 95% Confidence Interval. P-value: Statistical significance of beta coefficients for an interaction term before multiple testing correction. None of interaction terms were significant when UTP at 6-month and 18-month follow-ups were examined as well.

SA (Assessment)	ABCD Instrument (Assessment)	ABCD Variable	OR	L95	H95	P-value
Year 2	CBCL (Year 1)	cbcl_scr_07_stress_t	0.6	0.4	0.91	1.52E-02
Year 2	CBCL (Year 1)	cbcl_scr_syn_thought_t	0.57	0.36	0.91	1.70E-02
Year 1	CBCL (Year 1)	cbcl_scr_07_stress_t	0.6	0.4	0.92	1.74E-02
Year 1	CBCL (Year 1)	cbcl_scr_syn_thought_t	0.58	0.36	0.91	1.79E-02
Year 1	Personality/Temperament (Year 1)	Total.psychosis.symptoms	0.63	0.42	0.94	2.28E-02
Year 2	CBCL (Year 2)	cbcl_scr_07_ocd_t	0.65	0.44	0.95	2.72E-02
Year 2	CBCL (Year 2)	cbcl_scr_syn_thought_t	0.63	0.41	0.96	3.15E-02
Year 2	CBCL (Year 2)	cbcl_scr_07_stress_t	0.66	0.45	0.97	3.24E-02
Year 2	CBCL (Year 2)	bin_cbcl_scr_syn_thought_t	0.09	0.01	0.85	3.53E-02
Year 1	CBCL (Year 2)	cbcl_scr_07_ocd_t	0.66	0.45	0.98	3.84E-02
Year 1	CBCL (Year 1)	cbcl_scr_07_ocd_t	0.59	0.36	0.98	4.01E-02
Year 2	Personality/Temperament (Year 1)	Total.psychosis.symptoms	0.66	0.44	0.98	4.03E-02
Year 1	CBCL (Year 2)	cbcl_scr_07_stress_t	0.67	0.45	0.99	4.19E-02
Year 1	Personality/Temperament (Year 1)	Psychosis.severity	0.69	0.48	0.99	4.29E-02
Year 2	Cognition	Attention	1.94	1.02	3.69	4.32E-02
Year 2	CBCL (Year 1)	cbcl_scr_07_ocd_t	0.6	0.36	0.99	4.47E-02
Year 2	CBCL (Year 2)	cbcl_scr_syn_anxdep_t	0.62	0.39	0.99	4.59E-02
Year 1	CBCL (Year 2)	cbcl_scr_syn_thought_t	0.65	0.42	1	5.05E-02
Baseline	CBCL (Year 1)	cbcl_scr_07_sct_t	0.59	0.35	1	5.06E-02
Baseline	CBCL (Baseline)	cbcl_scr_07_sct_t	0.57	0.32	1	5.12E-02
Year 1	CBCL (Year 1)	cbcl_scr_07_sct_t	0.66	0.43	1.01	5.30E-02
Year 1	CBCL (Year 2)	cbcl_scr_syn_anxdep_t	0.63	0.39	1.01	5.40E-02
Year 2	CBCL (Year 2)	cbcl_scr_syn_totprob_t	0.59	0.34	1.01	5.40E-02
Baseline	Personality/Temperament (Year 2)	Mania	1.75	0.99	3.1	5.50E-02
Year 2	CBCL (Year 2)	cbcl_scr_syn_social_t	0.73	0.52	1.01	5.60E-02
Baseline	CBCL (Year 1)	cbcl_scr_syn_thought_t	0.61	0.36	1.01	5.64E-02
Year 2	CBCL (Baseline)	bin_cbcl_scr_syn_thought_t	0.2	0.04	1.04	5.64E-02
Year 2	CBCL (Year 2)	cbcl_scr_dsm5_depress_t	0.64	0.4	1.02	5.80E-02
Year 1	CBCL (Year 2)	cbcl_scr_dsm5_depress_t	0.63	0.4	1.02	5.81E-02
Baseline	CBCL (Baseline)	bin_cbcl_scr_syn_thought_t	0.12	0.01	1.1	6.03E-02
Year 1	CBCL (Baseline)	bin_cbcl_scr_syn_thought_t	0.2	0.04	1.07	6.04E-02

© 2024 Lee PH et al. *JAMA Network Open.*

Year 2	CBCL (Year 2)	cbcl_scr_syn_rulebreak_t	0.72	0.51	1.02	6.10E-02
Year 2	CBCL (Year 2)	cbcl_scr_syn_aggressive_t	0.73	0.53	1.02	6.15E-02
Baseline	CBCL (Year 1)	cbcl_scr_syn_attention_t	0.68	0.46	1.02	6.36E-02
Year 1	Cognition	Attention	1.84	0.96	3.51	6.44E-02
Year 2	Personality/Temperament (Year 1)	Psychosis.severity	0.71	0.5	1.02	6.60E-02
Year 2	CBCL (Year 2)	cbcl_scr_dsm5_conduct_t	0.71	0.49	1.02	6.64E-02
Year 1	CBCL (Year 2)	bin_cbcl_scr_syn_thought_t	0.12	0.01	1.17	6.83E-02
Year 2	CBCL (Year 1)	cbcl_scr_dsm5_adhd_t	0.68	0.44	1.03	7.04E-02
Year 1	CBCL (Year 1)	cbcl_scr_dsm5_adhd_t	0.68	0.44	1.04	7.16E-02
Baseline	Personality/Temperament (Year 1)	Total.psychosis.symptoms	0.64	0.39	1.04	7.41E-02
Year 2	CBCL (Year 1)	cbcl_scr_07_sct_t	0.68	0.45	1.04	7.53E-02
Year 2	personalitYear 2	Positive.urgency	0.64	0.38	1.05	7.59E-02
Baseline	CBCL (Year 2)	cbcl_scr_syn_social_t	0.71	0.48	1.04	8.00E-02
Year 1	CBCL (Year 2)	cbcl_scr_syn_social_t	0.74	0.53	1.04	8.22E-02
Year 2	CBCL (Year 2)_bin	bin_cbcl_scr_07_ocd_t	0.21	0.03	1.22	8.22E-02
Baseline	cbcl0_all	cbcl_scr_syn_attention_t	0.63	0.37	1.06	8.36E-02
Year 2	CBCL (Year 2)_bin	bin_cbcl_scr_dsm5_conduct_t	0.13	0.01	1.33	8.56E-02
Baseline	cbcl0_all	cbcl_scr_syn_totprob_t	0.58	0.31	1.08	8.66E-02
Year 1	CBCL (Year 1)	cbcl_scr_syn_attention_t	0.74	0.53	1.04	8.70E-02

© 2024 Lee PH et al. *JAMA Network Open.*

eTable 22. Multivariate Logistic Regression Results for Predicting Suicide Attempts (SAs) While Integrating Multiple Types of Substance Use Data in Addition to Smoking Tobacco Products[a]

Along with sociodemographic, family history, prenatal exposure, and significant correlates of childhood outcomes, both UTP and each of other substance use data (ie., alcohol sipping, drinking, cannabis use, and prescription drug use) were tested in the multivariate logistic regression model using SA as a dependent variable. Other covariates data are omitted for clarity.

Category	Predictor	Multivariate Logistic Regression				Likelihood Ratio Test	
		OR	95% CI	P-value	R^2 (%)	P-value	FDR
Substance Use	UTP	2.7	1.46-4.99	3.18	1.49E-03	0.64	2.81E-03
	Alcohol Sipping	1.55	1.14-2.13	2.75	5.90E-03	0.53	6.61E-03
	UTP	3.26	1.77-5.98	1.40E-04	0.89	4.18E-04	7.73E-03
	Alcohol Drinking	0.53	0.13-2.13	3.71E-01	0.07	3.41E-01	7.89E-01
	UTP	2.78	1.50-5.16	1.22E-03	0.66	2.45E-03	3.56E-02
	Cannabis	2.33	1.01-5.37	4.62E-02	0.25	6.09E-02	3.22E-01
	UTP	2.89	1.56-5.35	7.35E-04	0.71	1.61E-03	2.98E-02
	Prescription Drugs	1.98	0.70-5.67	2.00E-01	0.11	2.23E-01	5.89E-01

[a] All listed predictors were examined simultaneously in the regression model. Likelihood ratio tests were conducted by comparing the full regression model with the same model without the corresponding predictor. Alcohol sipping and drinking refer to the experience of sipping/tasting alcohols and at least a full cup of drinking. Predictors with likelihood ratio test FDR<5% are marked in bold. OR: Odds Ratio, CI: confidence interval, LR Test: likelihood ratio test. R2: Nagelkerke's Pseudo R2

© 2024 Lee PH et al. *JAMA Network Open.*
