# Supplementary material for: Use of Tobacco Products and Suicide Attempts Among Elementary School–Aged Children
Source: JAMA Netw Open. 2024 Feb 26;7(2):e240376. doi: 10.1001/jamanetworkopen.2024.0376 (PMC10897745; doi:10.1001/jamanetworkopen.2024.0376)
Supplement: Supplement 2. — Data Sharing Statement [file jamanetwopen-e240376-s002.pdf]

Data Sharing Statement

Lee. Use of Tobacco Products and Suicide Attempts Among Elementary School–Aged Children. *JAMA Netw Open*. Published February 26, 2024.
doi:10.1001/jamanetworkopen.2024.0376

Data
Data available: No

Additional Information
Explanation for why data not available: The present study used the NIH Adolescent Brain Cognitive Development Study, which is publicly available from the NIMH Data Archive through the data access application by each study group. All data and data dictionaries are available from the NIMH Data Archive with the permission.
